# Nanocardboard as a nanoscale analog of hollow sandwich plates

**DOI:** 10.1038/s41467-018-06818-6

**Published:** 2018-10-25

**Authors:** Chen Lin, Samuel M. Nicaise, Drew E. Lilley, Joan Cortes, Pengcheng Jiao, Jaspreet Singh, Mohsen Azadi, Gerald G. Lopez, Meredith Metzler, Prashant K. Purohit, Igor Bargatin

**Affiliations:** 1 Mechanical Engineering and Applied Mechanics, University of Pennsylvania, 220 South 33rd Street, 229 Towne Building, Philadelphia, PA 19104-6315 USA; 2 Singh Center for Nanotechnology, University of Pennsylvania, 3205 Walnut Street, Philadelphia, PA 19104 USA

## Abstract

Corrugated paper cardboard provides an everyday example of a lightweight, yet rigid, sandwich structure. Here we present nanocardboard, a monolithic plate mechanical metamaterial composed of nanometer-thickness (25–400 nm) face sheets that are connected by micrometer-height tubular webbing. We fabricate nanocardboard plates of up to 1 centimeter-square size, which exhibit an enhanced bending stiffness at ultralow mass of ~1 g m^−2^. The nanoscale thickness allows the plates to completely recover their shape after sharp bending even when the radius of curvature is comparable to the plate height. Optimally chosen geometry enhances the bending stiffness and spring constant by more than four orders of magnitude in comparison to solid plates with the same mass, far exceeding the enhancement factors previously demonstrated at both the macroscale and nanoscale. Nanocardboard may find applications as a structural component for wings of microflyers or interstellar lightsails, scanning probe cantilevers, and other microscopic and macroscopic systems.

## Abstract (web summary)

Sandwich structures such as corrugated cardboard offer low weight and high bending stiffness, but they are difficult to produce at the nanoscale. Here, the authors combine webbing and perforation to produce alumina ‘nanocardboard’ with ultralow areal density that recovers without damage from extreme deformation.

## INTRODUCTION

The sandwich structure, consisting of two planar face sheets connected by a webbing or foam core, is the optimally engineered design in applications that require plate-shaped structures of low weight and high stiffness^1,2^. Various types of sandwich structures, such as corrugated cardboard and honeycomb sandwich plates, have revolutionized many aspects of architecture, transportation, shipping, and packaging industries, but they are also commonly found in nature, including in plant leaves, microscopic diatom shells, crustacean shells, and skeletal bones^3–7^.

Sandwich plates offer significantly higher bending stiffness compared to a solid plate of the same mass because the two face sheets are offset from each other, increasing the effective moment of area, and because the shearing of the two face sheets is restricted by the separating core^1,2^. The corresponding enhancement in the bending stiffness, relative to a solid plate of the same mass, is known as the enhancement factor or shape factor^7,8^ and increases with increasing plate height and with decreasing face sheet thickness. However, it is typically limited to 10–100 at the macroscale because the face sheet thickness cannot be easily reduced below roughly one hundred micrometers for paper, or fractions of a millimeter for metals or fiber-reinforced resin^7–9^. Using films with nanoscale thickness could lead to much higher enhancement factors and enable large-area structures with nanoscale thickness that do not sag or bend under their weight, feature increased flexural resonance frequencies, or simply minimize the weight of plate-shaped structural components.

These characteristics could provide significant advantages to many technologies where weight is extremely important. For example, lightweight robotic microflyers require stiff, low mass density structural members for both the wings and body^10^. Another potential application is the Starshot lightsail, envisioned to travel at up to 20% of the speed of light in order to reach Proxima Centauri b in a couple decades^11^. Some of the critical material requirements include a mass density below 0.1 g m^−2^ (corresponding to a sail thickness of ~100 nm), the ability to sustain high temperatures, and a sufficient bending stiffness to control the shape (and thus the propulsion direction). While traditional sandwich structures offer a potential solution to such applications, they have yet to be scaled to the nanometer scale. The world’s thinnest published^12–17^ and commercial^18^ composite panels have an overall height on the order of one millimeter and element thicknesses of tens of microns. The main challenges in fabricating even smaller sandwich plates are that bonding face sheets to the webbing is difficult at micro/nanoscale and that ultrathin films tend to curl, buckle, or wrinkle spontaneously ^12,13,17^.

On the other hand, films of nanoscale thickness have recently been used as structural elements in novel cellular solids with a truss-like or lattice-like architecture^14,19–27^. These mechanical metamaterials typically exhibit higher Young’s moduli than their solid or random cellular material counterparts at low densities. Although the high Young’s moduli can potentially translate into plates with high bending stiffness, most truss-like mechanical metamaterials were fabricated in a cube-like, rather than a plate-like, shape, and were neither optimized for nor tested under bending loads. In contrast, we recently introduced the concept of a plate mechanical metamaterial, reporting nanometer-thick single-layer corrugated films that formed continuous plates and measuring their bending stiffness^28^.

**Fig. 1:**
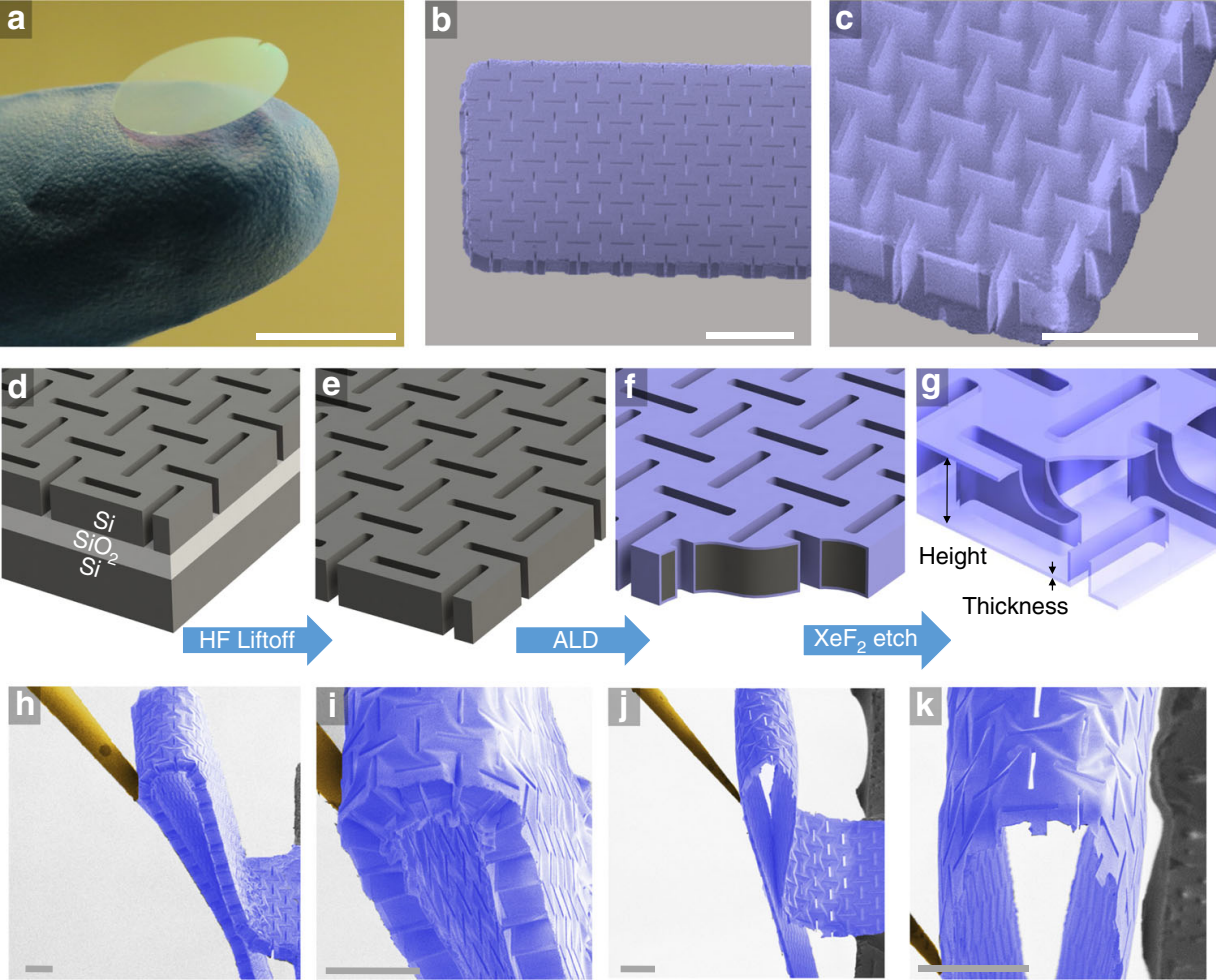
Images and schematics showing the nanocardboard plates. **a** Photograph of a large-area nanocardboard sample. **b** Scanning electron micrographs (false-colored) of a flat nanocardboard cantilever and **c** the detail of the basketweave webbing geometry. **d**–**g** Diagrams of the fabrication process for the nanocardboard structure with a basketweave webbing pattern. **h**–**k** Scanning electron micrographs (false-colored) of the recoverable sharp bending exhibited by basketweave nanocardboard plates with a thickness of 50 nm and a height of 50 μm (**h**, **i**) and 10 μm (**j**, **k**). All scale bars are 100 μm except in **a** where it is 10 mm. The images in **h**–**k** are representative of > 10 similar experiments

Yet, none of these previously reported mechanical metamaterial designs, including our corrugated plates^28^, exhibited the optimal spatial distribution of the material and the optimal scaling of the bending stiffness that is offered by the sandwich structure. In this paper, we present nanocardboard—a plate-shaped mechanical metamaterial that is a nanoscale analog of corrugated cardboard or web-core sandwich plates. Shown in Fig. 1a, our micro/nanofabricated nanocardboard plates have a microscale height, nanoscale thickness of face and webbing elements, and centimeter-scale lateral dimensions. However, the face sheets and the webbing of the nanocardboard differed from the wavy webbing of corrugated cardboard or the interconnected core of honeycomb sandwich structures. Instead, we used the webbing of spanning rectangular tubes (Fig. 1b–d) and face sheets that contained perforations matching the cross sections of the webbing tubes. This architecture resulted from simultaneous deposition of the webbing film and face sheets on a sacrificial mold, creating a monolithic hollow structure made from a single material with nanoscale thickness (25–400 nm).

## RESULTS

### Optimal design

The webbing/perforation pattern is critical to the mechanical characteristics of nanocardboard because the bending stiffness is largely determined by the tensile stiffness of the face sheets, which is reduced by any perforations. The geometries and mechanics of perforated planar sheets have been explored extensively in the literature^29–32^, providing many examples of face sheet geometries that can be adapted into corresponding nanocardboard designs. As further discussed in Supplementary Note 1 and Supplementary Fig. 3, we ran many numerical optimizations and ultimately chose the basketweave webbing pattern shown in Fig. 1 and Supplementary Fig. 1 for two reasons. First, it provided a combination of a relatively high tensile stiffness and the ability to accommodate large elongations^33^, which for nanocardboard, translates into a high bending stiffness and the ability to recover from extreme bending deformations. Second, it prevented the spontaneous wrinkling of the face sheets as long as the webbing pattern satisfied the no-straight-line condition (Supplementary Fig. 2), that is, any potential straight-line wrinkle/crease must intersect a webbing feature.

### Fabrication

The plates were microfabricated using the process described in detail in the Methods Section and Supplementary Methods. Briefly, photolithography and etching were used to etch tubular holes in a thin silicon mold, which was then conformally coated with alumina using atomic layer deposition (ALD). The alumina shell was released by dry etching the silicon out from the interior. While we focused our measurements on alumina plates up to 1 cm in size because they were sufficient for the characterization of bending and shear stiffness, nanocardboard plates can be made of any other conformally deposited material, and production could potentially be scaled to 6-inch wafer substrates producing square meters of nanocardboard on the timescale of a day. The mold can also be fabricated using other techniques such as two-photon stereolithography, although the maximum lateral sizes could be limited for such serial fabrication techniques.

The resulting nanocardboard samples could be handled by hand or tweezers as they are surprisingly robust with respect to bending and out-of-plane compression. Also, when immersed in water or acetone, the plates survived without showing any deflection or failure from the surface tensions of the drying meniscus (see Supplementary Fig. 6), in contrast to failure that is common in other thin-walled mechanical metamaterials ^28^.

### Recovery from extreme bending deformations

The thinnest nanocardboard plates we fabricated could sustain very sharp bending without catastrophic damage, as illustrated in Fig. 1h–k and Supplementary Note 4. This behavior of recoverable deformation with a brittle material has been observed for a few other architected micro/nanostructures^23,27,34,35^, but only under compressive loads. Sharp-bending recovery does not have a precedent in macroscopic sandwich plates or microscopic sandwich plates with continuous face sheets, which typically fail via the yield, fracture, or delamination of the face sheets, or irreversible buckling of the webbing^1,2^. For metal, paper, and plastic composite sandwich plates, the failure typically occurs when the radius of curvature is about two orders of magnitude larger than the height of the plate structure, and once a macroscopic sandwich plate forms a crease, it is irreversibly weakened and typically cannot recover the original flat shape and bending stiffness. In contrast, our nanocardboard plates sustained a radius of curvature down to just a few times the characteristic element size of the nanocardboard—the height of the plate or the period of the webbing, whichever is larger—and recovered without visible damage after such deformations (see Supplementary Movie 1). As discussed in Supplementary Note 4 and Supplementary Fig. 8, the nanocardboard plates can elastically recover without fracture or irreversible buckling because the local strains never exceed ~1%, which is below the typical yield strain of ultrathin ALD films^36^. In contrast, our simulations of hypothetical nanocardboard sandwich plates with continuous, unperforated face sheets showed > 4% strain under similar bending deformation (Supplementary Fig. 8h and 8i) while a solid uniform plate would experience even larger strains of > 10%. In these more traditional geometries, the bending-induced strains would fracture not only alumina but most other non-elastomer materials. In nanocardboard, the disconnected nature of the tubes allowed them to reorient with respect to neighboring tubes, and the nearby face sheet films reversibly buckled in response to the reorientation. Such a phenomenon is uncommon in other sandwich plates where the core is interconnected (e.g., expanded honeycomb) or continuous (e.g., foam). Based on these observations, the perforated nature of our nanocardboard is crucial for not only the monolithic fabrication process but also the observed shape recovery.

### Definition of bending stiffness of microscale sandwich plate

Bending stiffness, also known as flexural modulus, is one of the most important characteristics of architected plates since the plates are typically used to support out-of-plane loads. To determine the optimal geometry of the webbing, we performed extensive finite-element simulations using COMSOL and ABAQUS software packages (see Supplementary Note 3 and Supplementary Fig. 7). The results indicated that the bending stiffness, *D*, of basketweave nanocardboard was maximized when the length of the webbing rectangles was much larger than their width and the webbing rectangles were spaced as far as the no-straight-line rule allows. Both simulations and experiments showed that such a high-aspect-ratio basketweave pattern has *D* ≈ 0.3*D*_ideal_, where $D_{\mathrm{ideal}} = 1/2Eth^2/\left( {1 - \nu^2} \right)$ is the bending stiffness of an ideal theoretical sandwich plate with continuous face sheets, *E* and *v* are the Young’s modulus and Poisson ratio of alumina, *h* is the plate height, and *t* is the face sheet thickness^1^. These results are consistent with previous simulations of the tensile properties of sheets with basketweave perforations, in which the tensile stiffness was ~30% of that of a sheet without perforations^33^. Despite the orthogonal nature of the basketweave pattern, the bending stiffness of the plates was approximately isotropic, deviating by no more than 10–15% from the maximum value for different bending directions (see Supplementary Note 3 and Supplementary Fig. 7).

### Measurement of bending and shear stiffness

To characterize the mechanical properties of the nanocardboard structure, we measured the response of cantilevers to out-of-plane loads using an atomic force microscope (AFM). In general, for an out-of-plane end load *F* on a cantilever with length *L* and width *W*, the total deflection is caused by both the shearing and the bending deformations: $\delta _{{\mathrm{total}}} = \frac{{FL}}{{GhW}} + \frac{{FL^3}}{{3DW}}$, where *G* is the transverse shear modulus and *D* is the bending stiffness of the cantilever material^1^. For a solid cantilever that is much longer than it is thick (*L* ≫ *t*), the bending-induced displacement typically dominates, and the shear-induced deflection can be neglected. Previously reported microfabricated cantilevers were typically solid and long, and thus well described by the Euler-Bernoulli theory, with their spring constant depending on only the bending stiffness: $k_{{\mathrm{cant}}} = 3DW/L^3$. For solid cantilevered plates, the bending stiffness is given by $D_{{\mathrm{solid}}} = \frac{{Et_{{\mathrm{solid}}}^3}}{{12\left( {1 - \nu ^2} \right)}}$, where *t*_solid_ is the thickness of the solid cantilever, *E* is the elastic (Young’s) modulus of the cantilever material, and *v* is its Poisson’s ratio. Combining the two expressions results in the familiar formula for the spring constant of a high-aspect-ratio solid cantilevered plate: $k_{{\mathrm{solid}}} = \frac{{EWt_{{\mathrm{solid}}}^3}}{{4L^3\left( {1 - \nu ^2} \right)}}$.

**Fig. 2:**
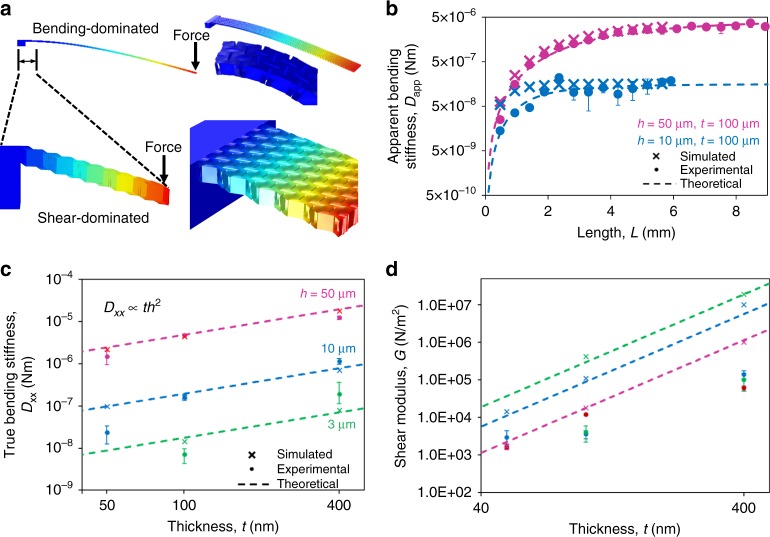
Schematic and plots of the cantilever deflection and characterized properties. **a** Schematic of two different modes of cantilever deflection: bending-dominated deformation of very long cantilevers (top), and shear-dominated deformation of shorter cantilevers (bottom). The insets provide different angled views and magnification to show the bending and shear characteristics. The bottom image shows the staircase pattern of shear deformation that is caused by the disconnected webbing. **b** Plot of experimental and finite-element-simulated *D*_app_ versus lengths *L* for two example cantilevers. The experimental data are calculated from the spring constant measured with AFM probing. As the cantilever length increases beyond the critical length *L*_*c*_~ 1 mm, the apparent bending stiffness saturates, indicating the transition from the shear-dominated to the bending-dominated regime. The data points represent two separate cantilevers. **c** *D*_xx_ and **d** *G* extracted from curve fitting, such as that in **b**, along with the theoretically expected scaling trends. Error bars are provided as 1 standard deviation for the experimental data points. The data were fitted from 9 cantilevers and is representative of ~100 other cantilevers

However, hollow cellular plates can have a very low shear modulus and can, therefore, exhibit shear-dominated deflection even in moderately long cantilevers^1^. As a result, naively using the standard Euler-Bernoulli relationship $D = k_{{\mathrm{cant}}}\,L^3/3W$ gives the true bending stiffness of a cantilever plate only for extremely long cantilevers; for less long cantilevers, it instead gives the apparent bending stiffness. Using the more accurate Timoshenko beam theory, the apparent bending stiffness can be written as $D_{{\mathrm{app}}} = L^2/\left( {\frac{{L^2}}{{D_{{\mathrm{xx}}}}} + \frac{3}{{Gh}}} \right)$, where *D*_xx_ (with units of N m) is the true bending stiffness of the sandwich plate along the length of the cantilever and *G* (N m^−2^) is its transverse shear modulus (details in Supplementary Note 5). In short cantilevers (Fig. 2a bottom), shear dominates the deflection and the apparent bending stiffness increases with the cantilever length as $D_{{\mathrm{app}}} = GhL^2/3$. However, above a certain critical length^37^, roughly $L_{\mathrm{c}}\sim \sqrt {\frac{{3D_{{\mathrm{xx}}}}}{{Gh}}}$, the total deflection is dominated by bending deformation and *D*_app_ saturates at the true bending stiffness, $D_{\mathrm{xx}} \approx 0.3 \times D_{{\mathrm{ideal}}} \approx 0.15Eth^2$ (Fig. 2a top).

To determine the bending and shear stiffness experimentally, we fabricated nanocardboard cantilevers of constant width, *W* ≈ 500 μm, and lengths *L* ranging from < 0.5 to > 10 mm, as detailed in Supplementary Methods. The load vs. displacement curves were measured with an AFM, providing *k*_cant_ and thus the experimental value of *D*_app_ (see Supplementary Figs. 4 and 5). Figure 2b shows examples of the apparent bending stiffness *D*_app_ for different points along the length of two cantilevers, illustrating the transition from shear-dominated to bending-dominated deformation. There was good agreement between the experimental and simulated data points, obtained from full-size COMSOL finite-element models (see Supplementary Note 3). The experimental data from the cantilevers were then fit to the Timoshenko-theory formula $D_{{\mathrm{app}}} = \left( {\frac{1}{{D_{{\mathrm{xx}}}}} + \frac{3}{{L^2Gh}}} \right)^{ - 1}$, resulting in the bending and shear stiffness shown in Figs. 2c, d. The experimental and simulation-based data generally matched each other within the margin of error. As detailed in Supplementary Note 2, the larger error bars observed for some of the *h* = 3 μm and *t* = 50 nm data can be explained by the higher experimental noise present and the low number of shear-dominated data points in the testing of the longer *L* > 3 mm cantilevers with relatively low spring constants. In addition, the experimental values for the shear stiffness deviated from the simulated values for the largest thickness of *t* = 400 nm. This discrepancy is likely due to the effect of imperfectly rigid cantilever clamping since the discrepancy only appeared for the measurements of the shortest and thickest samples.

### Analytical model of nanocardboard

In addition to the finite-element simulations, we also developed a simplified bi-rod-derived model, which provides an analytical insight into how the true bending stiffness and the shear modulus are expected to scale with the geometric parameters of the webbing (see Supplementary Note 5 and Supplementary Figs. 9 and 10 for details). The model consists of two face sheets connected by cylindrical webbing, all of which are capable of elastic extension, shearing, and bending. The governing equations obtained via the balance of forces and moments accurately describe the mechanics of the outer plates and the web under the assumptions that the loading and boundary conditions are homogeneous along one direction. A novel feature of the model is that we account for the granularity, or discontinuous nature, of the webbing pattern, which is crucial for getting the correct scaling laws. As can be seen in the bottom of Fig. 2a, the face sheets of nanocardboard do not deform as a smooth arc but rather in the staircase fashion, and our analytical model accounted for this granularity by considering the webbing spacing/period, *s*.

The bi-rod-based analytical model gives accurate predictions for the trend/scaling of the bending stiffness and shear transverse modulus with the geometric parameters of the webbing, although the quantitative predictions are less accurate since the two-dimensional analytical model cannot capture the full complexity of the three-dimensional basketweave webbing. According to the model, *D*_xx_ is predicted to scale as *th*^2^ and *G* as *t*^3^/(*s*^2^*h*). The critical length *L*_*c*_ is predicted to scale as $L_{{\mathrm{c}}} \propto \sqrt {D/Gh} \propto sh/t$. As shown in Figs. 2c, d, the experimental and numerical data points generally match the scaling predicted by the bi-rod-derived model except for the experimental discrepancies as discussed above.

### Comparison of nanocardboard stiffness

**Fig. 3:**
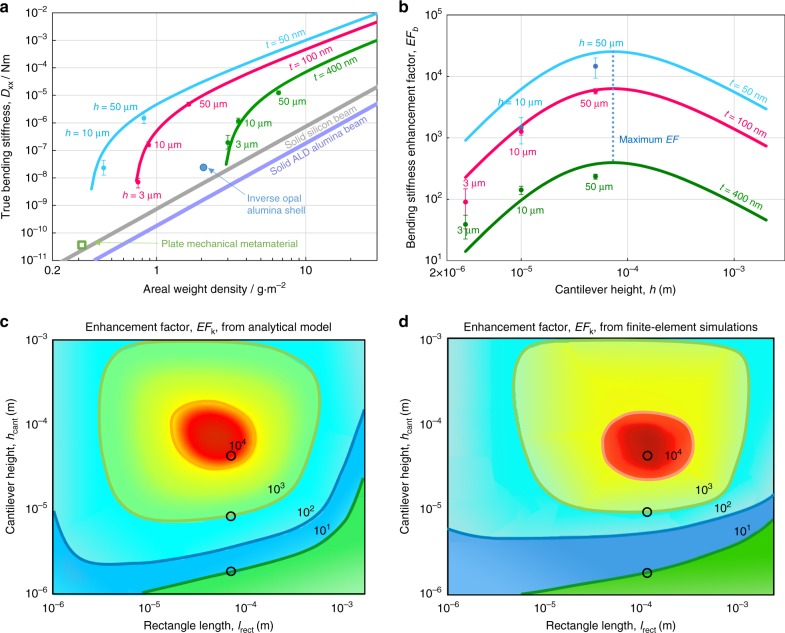
Comparisons of the bending stiffness and spring constant enhancement factors (EF). **a** Comparison of the bending stiffness and areal density of the nanocardboard structure to other plate materials. The nanocardboard bending stiffness is provided as experimental data points and theoretical trend lines, the same as those in Fig. 2. The green square and blue circle data points are for plate-like mechanical metamaterials: ultrathin corrugated alumina^28^ and inverse-opal alumina shell^39^. The theoretical stiffness of standard materials, silicon and alumina, are shown as baselines. **b** Enhancement factor for the bending stiffness of nanocardboard versus the cantilever height for the experimentally used basketweave parameters (*l*_rect_ = 50 μm, *w*_rect_ = 5 μm, *g* = 20 μm). **c** Density and contour plots of the enhancement factor for the spring constant, which considers both shear and bending deformations, versus the plate height and the webbing rectangle length. The plot is based on the analytical model described in the Supplementary Note 5 and assumes a cantilever length *L* *=* 9 mm and thickness *t* = 50 nm. The three open circles indicate the parameters used in experiments. **d** Same as (**c**) based on the interpolated results of finite-element simulations. The raw results of finite-element simulations are available in Supplementary Fig. 11c. Error bars are provided as 1 standard deviation for the experimental data points. The data in **a** and **b** are the same as Fig. 2c

Figure 3a compares the bending stiffness of the nanocardboard structure to previously reported materials of similar micron-scale height and millimeter-scale lateral dimensions. Analogous to Ashby charts that compare elastic modulus to density for bulk materials^1,27,38^, this figure plots the natural figures of merit for lightweight plate-like materials: true bending stiffness versus areal mass density. To provide a simple baseline, Fig. 3a also includes the bending stiffness of solid silicon and ALD alumina plates, for which $D_{{\mathrm{solid}}} = \frac{{Et^3}}{{12\left( {1 - \nu ^2} \right)}} = \frac{{E\,{\mathrm{AD}}^3}}{{12\left( {1 - \nu ^2} \right)\rho _{\mathrm{s}}^3}}$, where AD is the areal density and *ρ*_*s*_ is the standard volumetric density of the solid silicon or alumina.

At the lightest end of the graph, below 1 g m^−2^, the 50-nm thick plates outperform our previously reported ultralight corrugated plate mechanical metamaterials^28^. The taller versions of nanocardboard are also much stiffer than another reported alumina-based plate-shaped metamaterial that used the inverse-opal microarchitecture^39^. In addition to engineered plate materials, nature also provides an example of a nano/microscale sandwich structure in the silica skeleton of diatoms^6,24,25,40,41^. To date, the bending stiffness of this diatom shell has not been directly measured, though the reported Young’s modulus of the biosilica composite (~36 GPa or less)^41,42^ suggests that the diatom shell is more compliant than the ALD nanocardboard.

### Optimization of stiffness and spring constant

Following the literature on macroscopic sandwich plates and other hollow structures^7,8^, we can define the enhancement factor or shape factor as the ratio of the bending stiffness of the nanocardboard sandwich structure to that of a solid beam with the same areal density. For macroscale structured beams and plates, including sandwich plates, the maximum practical enhancement is typically less than 100^7,8^. This limitation is not intrinsic to the sandwich structure since the enhancement factor scales with the plate height and inversely with the face sheet thickness^2,37,43^. Rather, these practical limits are determined by the minimum achievable thickness of the face sheets, the cell size of the core material, the added weight of the adhesive, material fracture strength, or machining limits for the cores if they are brazed to the face sheet. The nanocardboard structure offers a unique platform to overcome these limitations because the face sheet thickness can be scaled down to tens of nanometers while the glue is avoided altogether since the entire structure is created in a single deposition step. Based on the analytical optimization shown in Supplementary Note 5 and Supplementary Fig. 11, the maximum bending stiffness enhancement factor for nanocardboard was achieved when the weight of the core elements equaled the weight of one face sheet, which enhanced the bending stiffness by more than four orders of magnitude for the geometric parameters we used in experiments.

Although one can, in principle, increase the bending stiffness indefinitely by increasing both the height and webbing spacing/scale, the resulting cantilever will become extremely soft with respect to shear displacements. In typical applications, shear stiffness should be optimized concurrently with the bending stiffness to maximize the overall spring constant of the cantilevered plate. As shown in Figs. 3c, d, the maximum enhancement of the spring constant EF_k_ (relative to a solid plate of the same weight) is achieved at the optimal values of the height and the webbing rectangle length, which are proportional to the geometric mean of the cantilever length and the film thickness, $\sqrt {Lt}$ (see Supplementary Note 5 for details). For these optimal parameters, the enhancement exceeds four orders of magnitude. As can be seen from Figs. 3c, d, the results of the analytical model and finite-element simulations agree, showing that for the specific cantilever length of 9 mm and film thickness of 50 nm, the 50-micron-tall nanocardboard plate achieves nearly the optimal enhancement factor in our experiments. We note that this optimal design analysis can be easily extended to other lengths and thicknesses, as well as from cantilevered plates to doubly clamped plates, membranes clamped on all sides, or other boundary conditions.

## DISCUSSION

In summary, this work introduces nanocardboard as a new ultralight nanometer-thick plate mechanical metamaterial with exceptional flatness, stiffness, and ultralow areal density (< 0.5 g m^−2^). The experimental results are well supported by finite-element simulations and a bi-rod-derived analytical model that correctly predicts the scaling of the bending stiffness and shear modulus versus plate thickness *t* and sandwich height *h*. Even after taking into account the shear, the nanocardboard plates with optimal webbing parameters offer spring constants that are four to five orders of magnitude larger than that of solid plates of the same areal weight.

Promising applications of nanocardboard include the wings of ultralight microflyers and hollow AFM probes. As a wing material for microflyers, nanocardboard can exhibit a low areal density while maintaining a relatively high bending stiffness and a high flexural resonant frequency, a combination of characteristics that is challenging to achieve in typical polymer film wings. In addition, the extremely low weight and high thermal insulation of nanocardboard plates enable microflyers based on new propulsion principles such as the photophoretic forces, also known as Knudsen or radiometric forces^44,45^. The same combination of properties makes nanocardboard an excellent mechanical substrate for a lightsail that can be used for interstellar travel^11^. As an AFM probe material, nanocardboard can provide high stiffness and frequency for sensitive measurements/imaging while also reducing the quality factor to enable high frame-rate scanning. Finally, as a chemical sensor it can offer an extremely high surface area in combination with high robustness and a high flexural resonance frequency, allowing sensitive and fast measurements^46^. Other potential applications include acoustic metamaterials, high-temperature thermal insulation, and other micromechanical systems for vacuum, gas, and liquid environments.

## METHODS

### Silicon mold fabrication

Silicon-on-insulator wafers were coated with a hard mask of SiO_2_ and Si_3_N_4_ via plasma-enhanced chemical-vapor deposition. The photomask was spin coated (Shipley Microposit S1818 resist) and exposed (Suss MicroTec, MA6 Gen 3, 300–500 mJ/cm^2^) to provide the webbing pattern and chip outlines (13-mm circles). Wafers were developed in MF-319 (Shipley Microposit) for 1–1.5 min and heated on a hotplate at 115 °C for 1 min. The webbing and outline pattern was transferred into the hard mask through CHF_3_/O_2_ reactive ion etching (RIE) (Oxford 80 Plus). The pattern was then transferred into the silicon via deep reactive ion etching (SPTS) with SF_6_ and C_4_F_8_.

### Silicon mold removal from wafer

To remove the chips, wafers were immersed upside down in a bath of 49% hydrofluoric acid for > 1 h to etch the oxide. Some of the chips released with careful rinsing with deionized water. The remaining chips were removed by carefully inserting a blade between the chip and the wafer inside a water bath.

### Atomic layer deposition

The alumina was deposited using Cambridge Nanotech S200 ALD (250 °C with a pulse of H_2_O vapor for 0.015 sec, delay of 5 sec, a pulse of tetramethylaluminum for 0.015 sec, and a delay of 5 sec) on chips that were taped to a custom glass carrier. The final deposited thickness of amourphous alumina was measured with spectral reflectometry (Filmetrics, F40 model).

### Laser machining of cantilevers and mounting

Alumina-coated chips were laser micromachined into individual cantilevers of 2–12 mm length and 0.5 mm width by cutting the outline of each cantilever (IPG IX280-DXF green laser, 50% power, 100 kHz rep rate, 1 to 250 passes for complete etch through). Machined cantilevers were mounted on glass slides with UV-curing epoxy (Loon Outdoors, High Viscosity).

### Etching of silicon mold

The silicon mold was etched with XeF_2_ vapor (Xactics/SPTS), leaving only the hollow nanocardboard structure. The etching (total cycles of 100–200, 60 sec each, 2 Torr vapor) was completed when the nanocardboard became optically translucent, and the dark silicon region had clearly disappeared.

### AFM Characterization

In order to characterize the spring constant of cantilevers, we used an atomic force microscope (AFM) (Asylum MFP-3D) at room temperature and commercial AFM probes (calibrated via the Sader method). A force-displacement measurement consisted of the reaction displacement of the AFM probe as it moved through a z-displacement of 10 μm when in contact with the nanocardboard cantilever. Measurement curves were obtained along the length of the cantilever from the base to the tip. We fitted a line along the contact portion of the force-displacement curve to calculate the spring constant and the corresponding apparent bending stiffness *D*_app_ at each point along the length of the cantilever.

## Electronic supplementary material

Supplementary Information

Peer Review File

Description of Additional Supplementary Files

Supplementary Movie 1

## Data Availability

All relevant data and code are available from the authors upon request.
