# Supplementary material for: Nanocardboard as a nanoscale analog of hollow sandwich plates
Source: Nat Commun. 2018 Oct 25;9:4442. doi: 10.1038/s41467-018-06818-6 (PMC6202357; doi:10.1038/s41467-018-06818-6)
Supplement: Supplementary file 1 — Supplementary Information [file 41467_2018_6818_MOESM1_ESM.pdf]

Supplementary Information for:

Nanocardboard as a nanoscale analog of hollow sandwich plates

by Lin et al.

Supplementary Figures

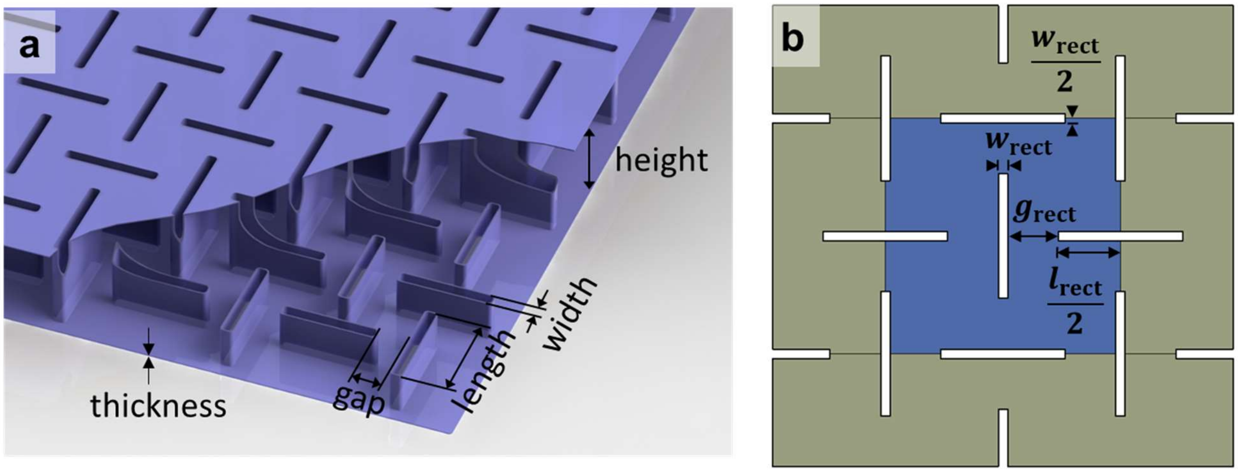

Supplementary Figure 1: a) Geometric parameters of the nanocardboard plate, including thickness, gap between webbing, length of webbing rectangle, width of webbing rectangle, and plate height. The gap was designed as 20 µm (measured 17.49-18.6 µm), the length designed as 50 µm (measured 50.9-52.39 µm) and the width designed as 5 µm (measured as 5.31-6.25 µm). The deposited thicknesses were nominally 50, 100, and 400 nm, and measured to be 49-53, 90-100, and 400-460 nm, respectively. The plate heights were determined directly from the thickness of the device layer of the silicon-on-insulator wafers used in fabrication: nominally 3, 10 and 50 µm, though not separately measured. b) Top-down schematic of (a) showing the geometric parameters within a single unit cell (dark blue).

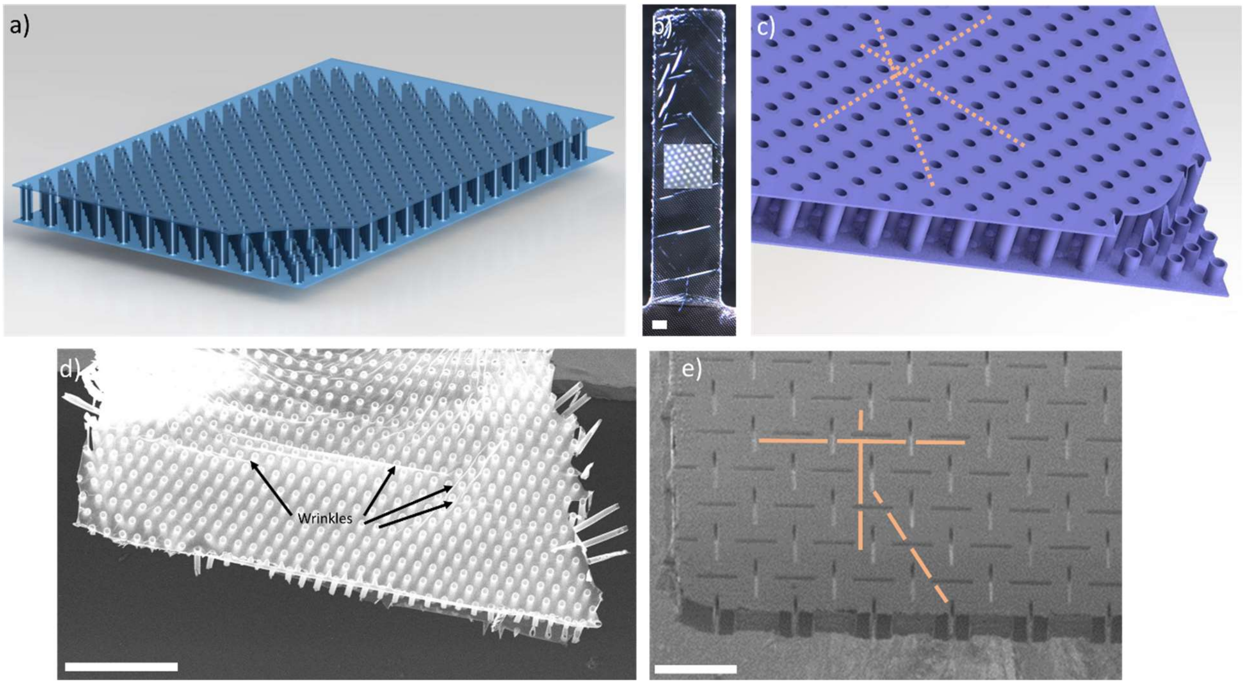

Supplementary Figure 2: Initial nanocardboard design that was limited by wrinkling and improved basketweave design. a) Nanocardboard with the webbing of a hexagonally periodic array of cylinders. b) Wrinkling of the face sheet (thickness ≤ 100 nm) on a cantilever under optical microscopy. We observed similar wrinkling in >6 samples. c) Schematic showing straight lines that go through the face sheet without intersecting any webbing cylinders. d) Scanning electron micrograph (SEM) of experimental observations of wrinkling along the 0°, 60° and 120° directions of the hexagonal lattice. e) SEM of basketweave pattern on a cantilever where straight lines are necessarily discontinuous due to the rectangular webbing. Scale bars are all 100 μm.

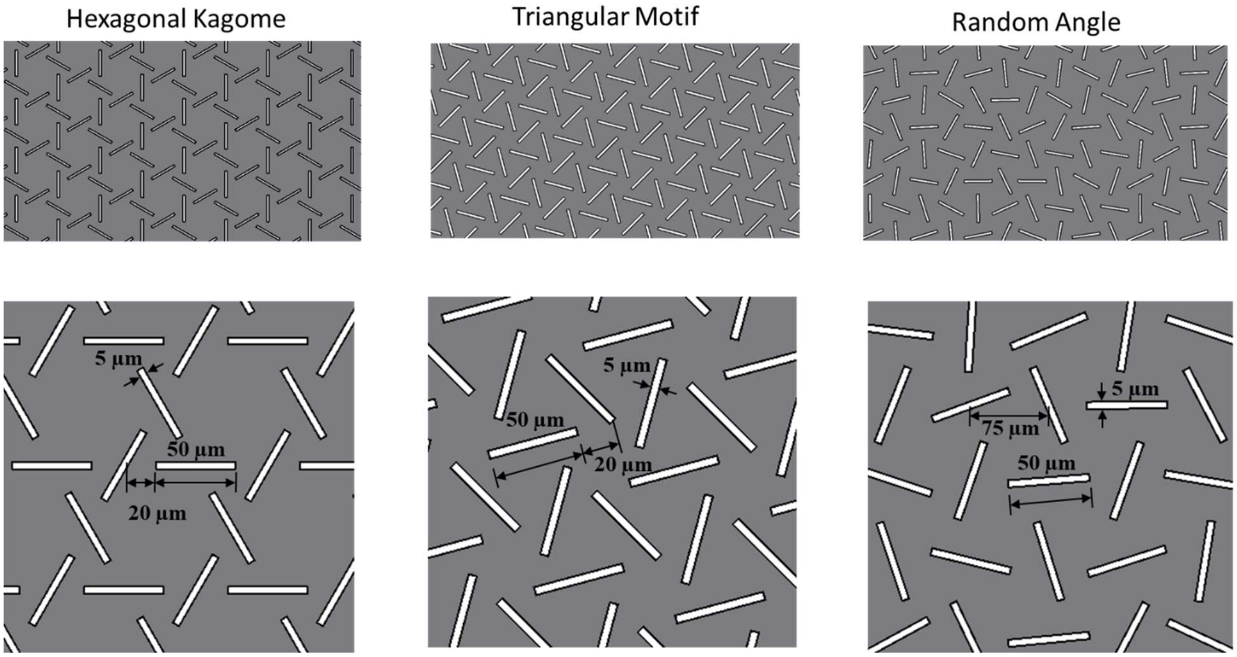

Supplementary Figure 3: Schematics of the simulated perforation/webbing patterns: hexagonal kagome, an analogous triangular motif, and randomly-oriented square basketweave. These patterns were specifically chosen because they were previously investigated, satisfied the no-straight-line rule and represented the three shapes that can be tessellated to fill a plane – a regular hexagon, regular triangle and regular square.

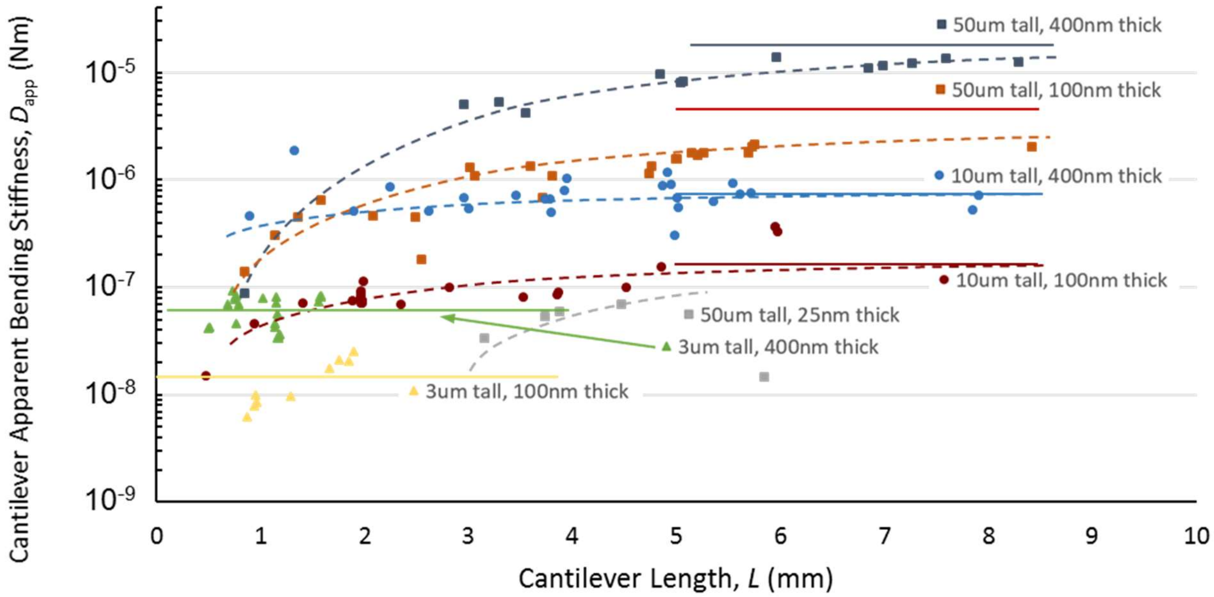

Supplementary Figure 4: Plot of the apparent bending stiffness D_{app} *vs. cantilever length* L *for a variety of different experimental cantilevers displaced at the cantilever tip. The dotted lines are meant to guide the eye, and the straight horizontal lines are roughly 30% of the theoretical sandwich plate bending stiffness, as discussed in the main text. The scatter of the experimental data due to imperfections of nanocardboard plates can be observed. Each data point represents a separate cantilever.*

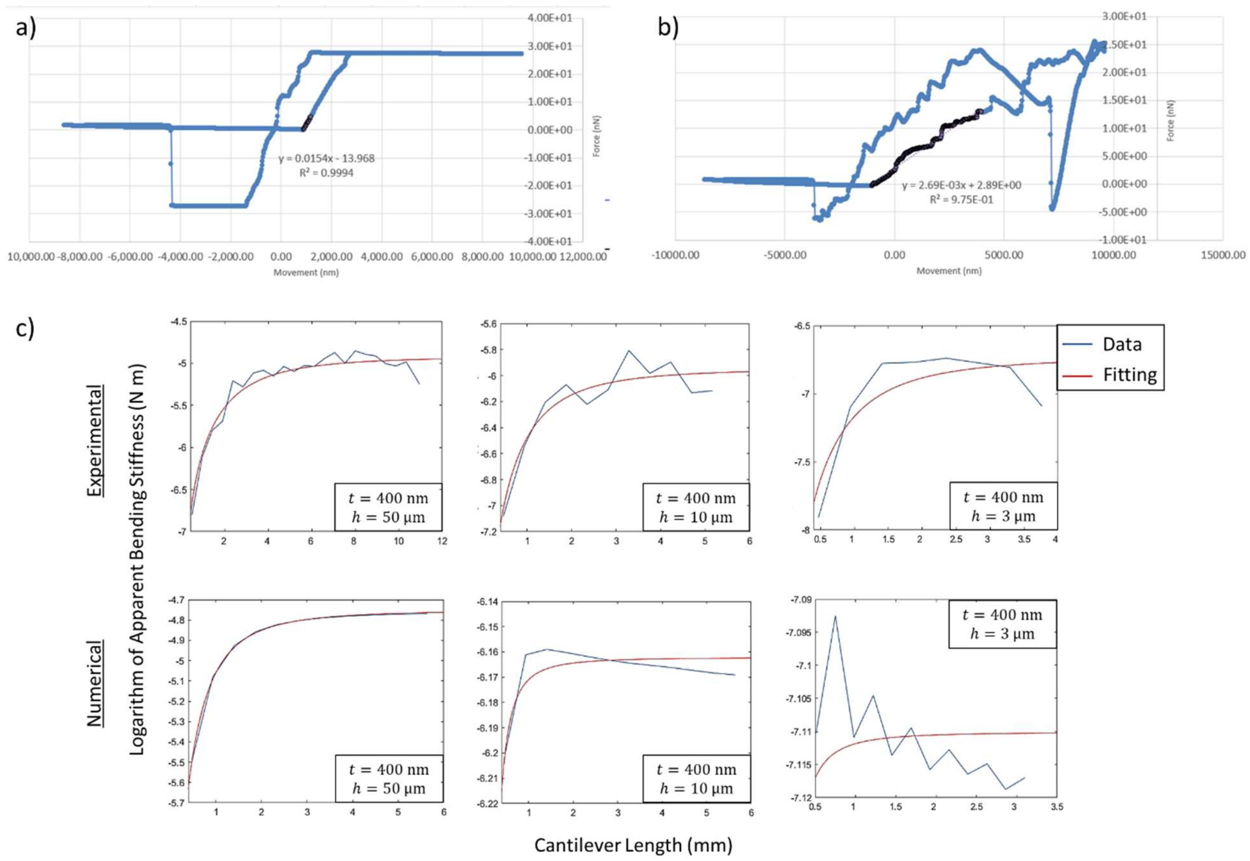

Supplementary Figure 5: Experimental and numerical simulation curves showing the mechanical response of cantilevers. a) and b) Example graphs of the force-displacement curves obtained from atomic force microscope (AFM) mechanical testing of nanocardboard cantilevers at different locations. The thickness, height and cantilever length for these images were: a) 50 nm, 50 µm, 1.47 mm (near the base) and b) 50 nm, 50µm, 5.64 mm (near the tip). The blue lines are the data obtained from the testing, and the black lines are the regions that were hand-selected to be fit. The displayed equation of the fit line gives the spring constant of the combined cantilevers (atomic force microscope probe plus nanocardboard cantilever). c) Fitted data for the along-the-length measurements. These graphs show the measured apparent bending stiffness in logarithmic units of Nm versus the measured length in millimeters. The solid black line represents the experimental data points, and the solid red line represents the numerical fitting of this data. The equations of the fitted lines were used to extract the values shown in Figs. 2 and 3 of the main text. Each graph in this figure is a measurement for a different cantilever.

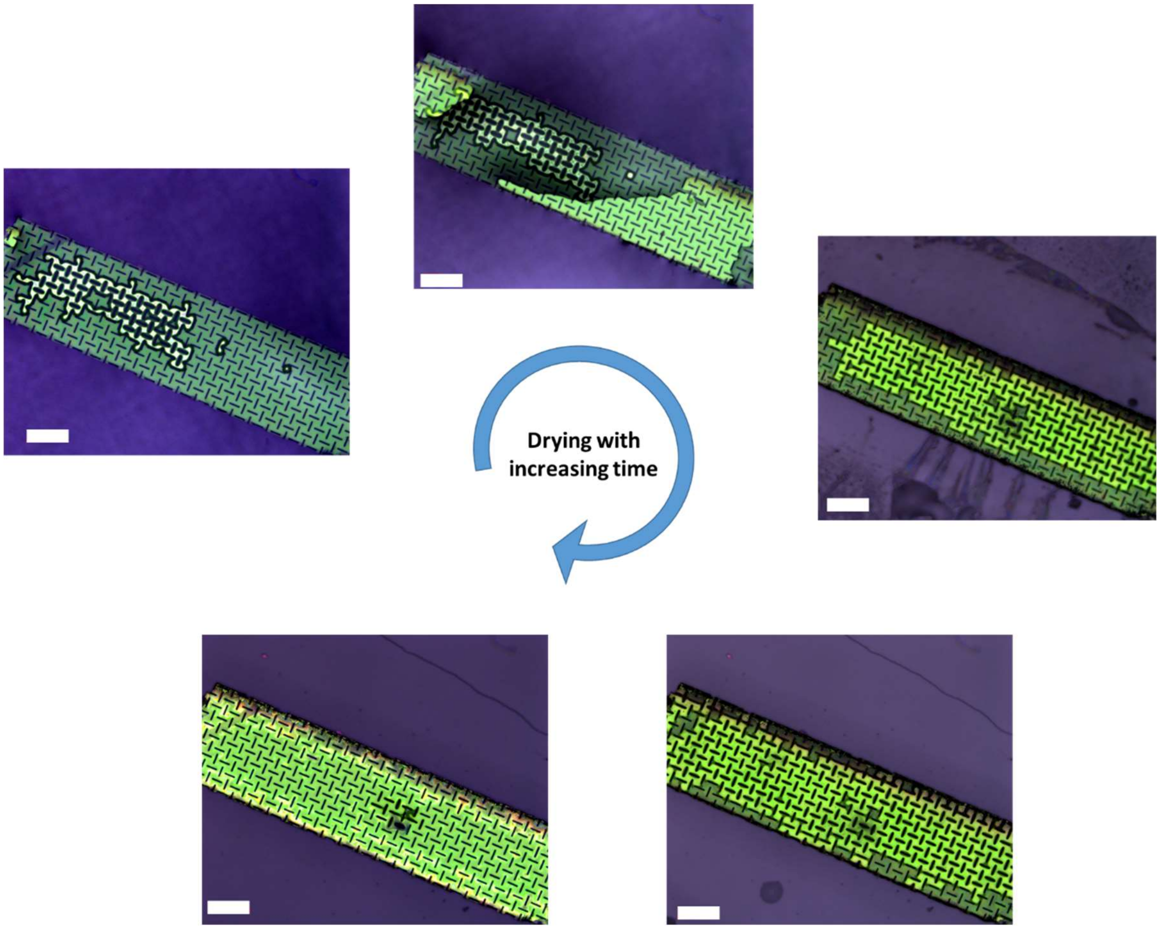

Supplementary Figure 6: Series of 5 optical microscope snapshots while acetone was drying from between the face sheet spacing of a nanocardboard cantilever. The darker green represents acetone within the cantilever, and the lighter green is the color without any liquid. The approximate time lapse for these five images is 6 minutes. We determined that the nanocardboard structure did not fail due to liquid meniscus tension since the final image shows the pattern intact without the discoloration characteristic of fracture. All scale bars are 200 µm. These images are for a single sample are representative of the results in 2 other samples.

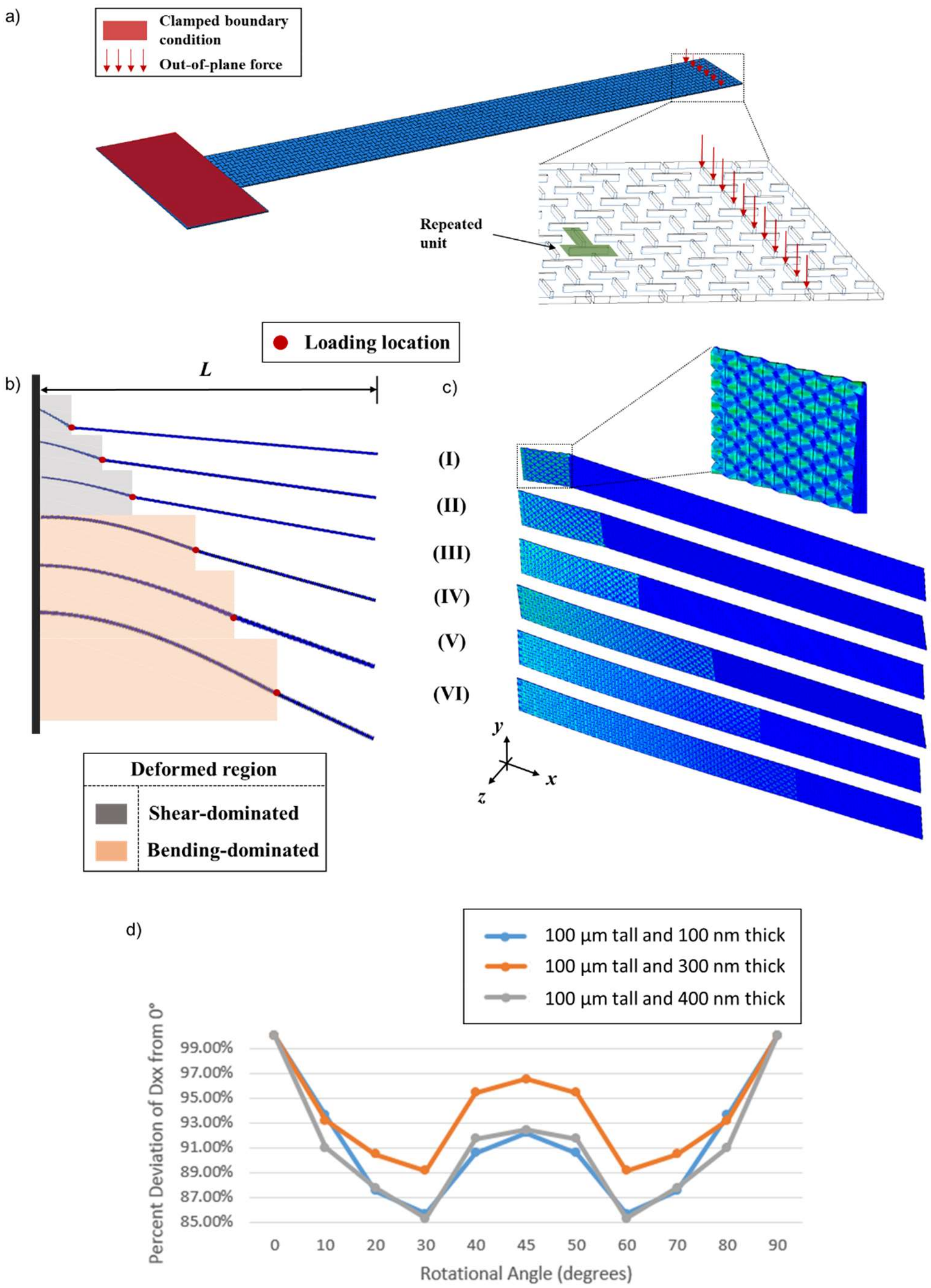

Supplementary Figure 7: a) Initial configuration, boundary and loading conditions of the finite element model. b) and c) Comparison of the deformed shape configuration of the nanocardboard by varying the loading location - (b) side view and (c) perspective 3D view. d) Change in bending stiffness for different orientations of the basketweave pattern with respect to the length of the beam direction.

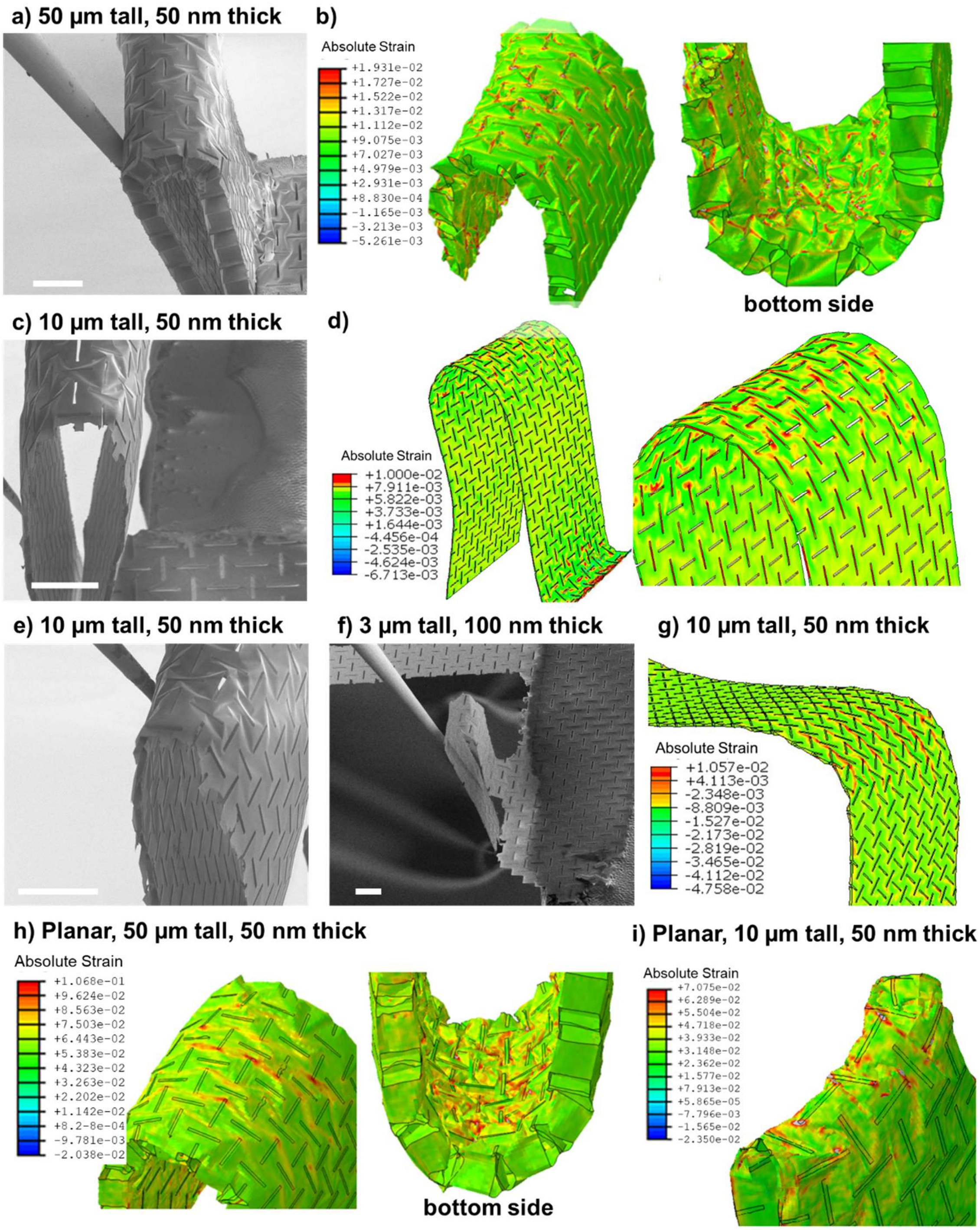

Supplementary Figure 8: SEM and finite-element (FE) simulation images of the bending of nanocardboard cantilevers which fully recovered to the initial position after testing. a) and b) Cantilever with 50 µm height and 50 nm thickness. c) and d) Cantilever with 10 µm height and 50 nm thickness. e) SEM of another bending test of a cantilever with 50 µm height and 50 nm thickness. f) and g) SEM and FE image of a bending test of a cantilever with 3 µm height and 100

nm thickness. h) and i) FE simulation images showing higher strain for structures with planar, instead of perforated, face sheets. Scale bars are all 100 μm. The SEM images are representative of >10 similar experiments.

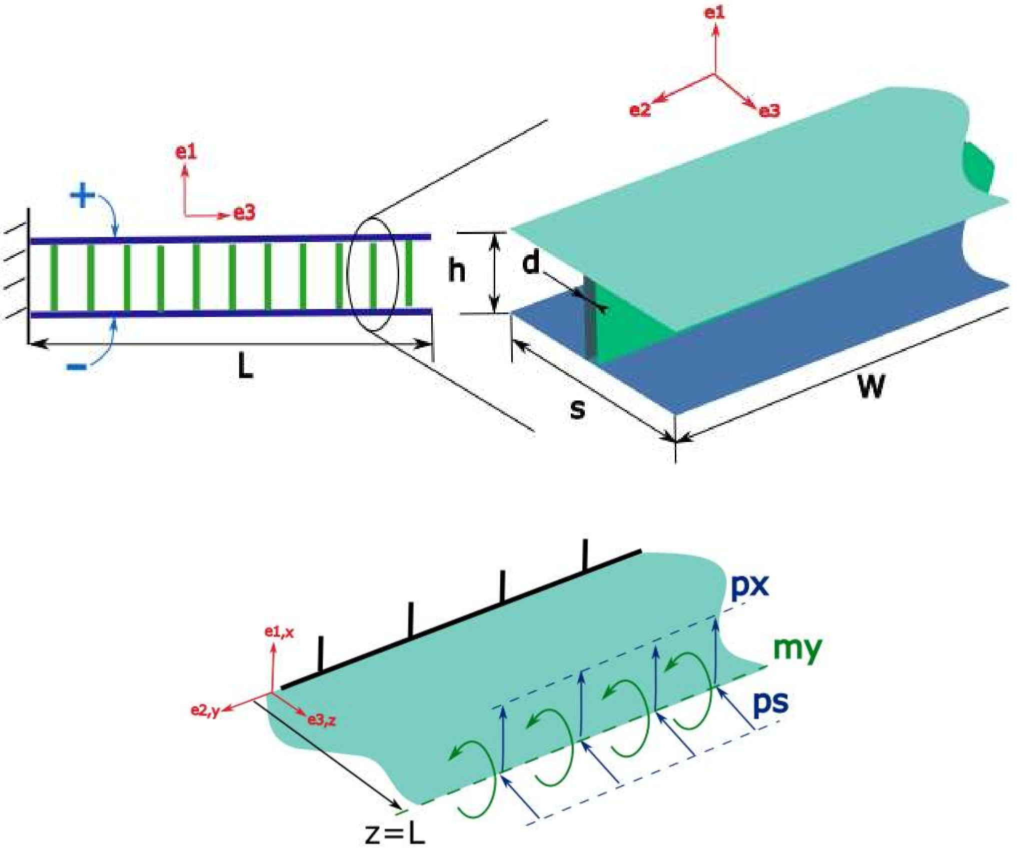

Supplementary Figure 9: Geometry of the theoreitcal bi-rod (top) and a cantilever plate bent by constant force $p_x\boldsymbol{e}_1 - p_s\boldsymbol{e}_3$ and moment $m_y\boldsymbol{e}_2$ at the edge $z = L$ (bottom).

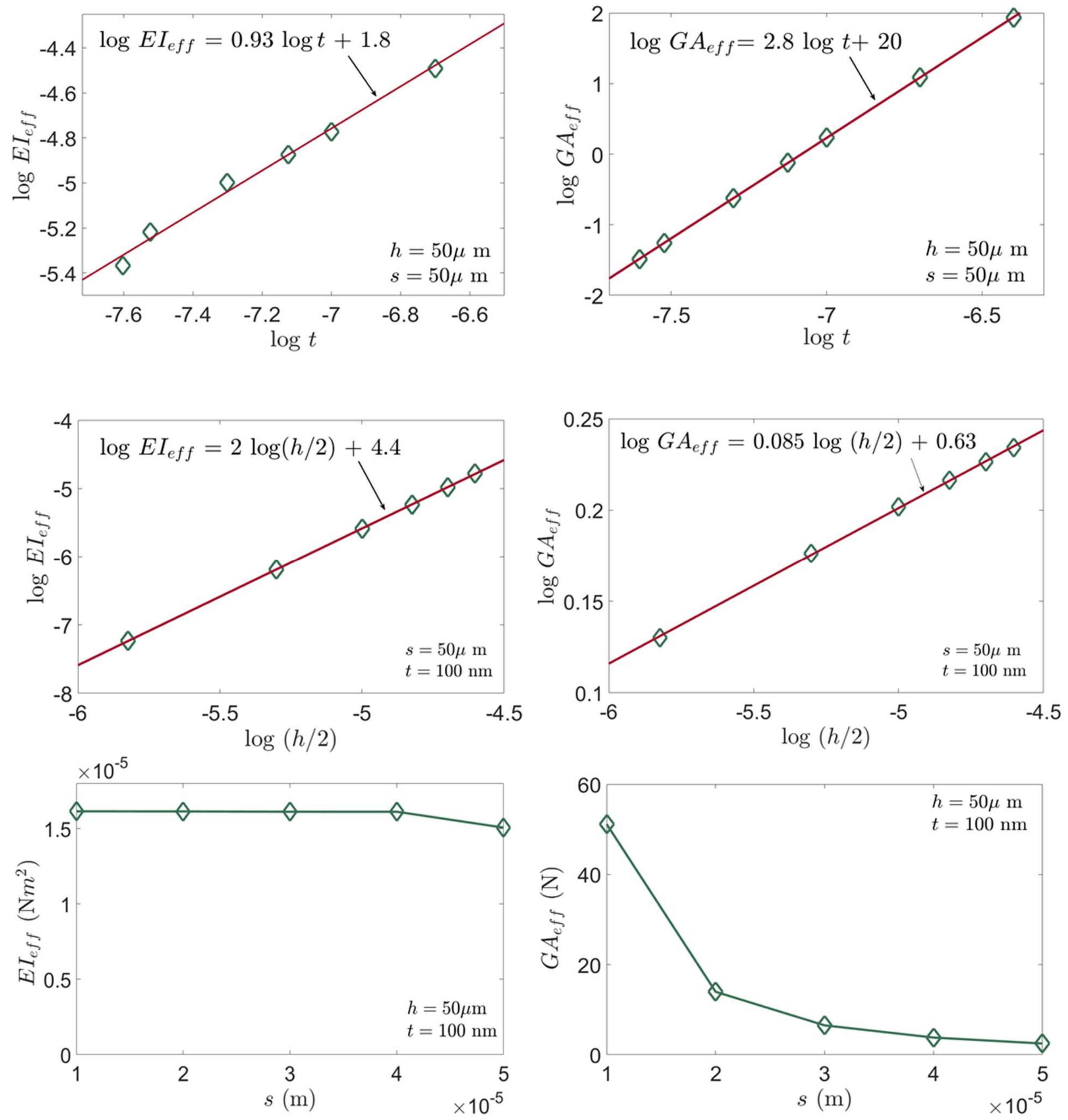

Supplementary Figure 10: Variation of bending stiffness EI_{eff} *and shear stiffness* GA_{eff} *with* t*, (a) and (b),* h*, (c) and (d), and* s*, (e) and (f).*

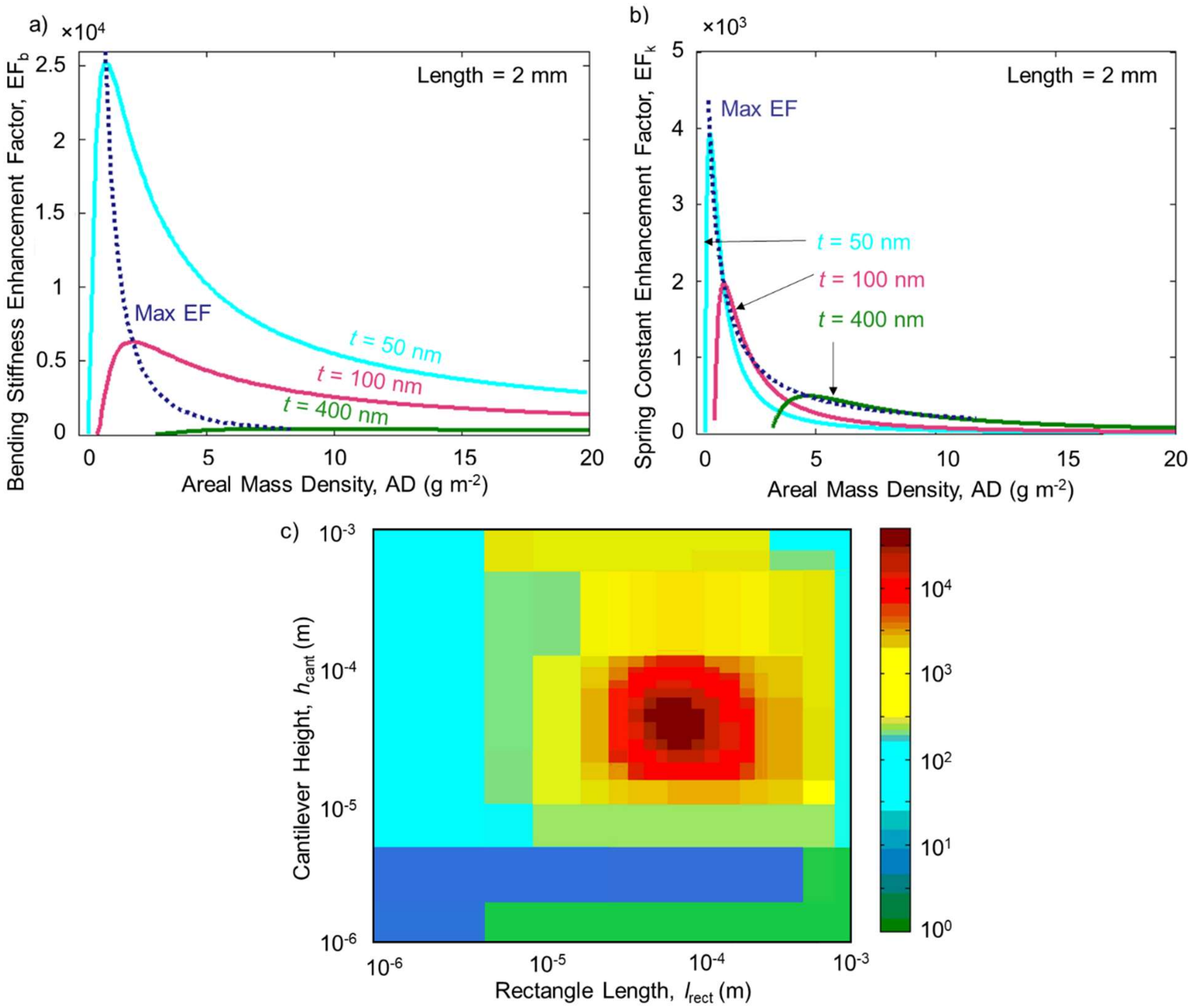

Supplementary Figure 11: Plots of the enhancement factor for different variables. a) The optimal bending stiffness enhancement factor plotted vs. the areal mass density of a beam for three different thicknesses t*. b) The same plot as a) though for the optimal spring constant enhancement factor. Note that for both a) and b), these analyzed beams were 2 mm long and 0.5 mm wide. c) Color density plot of the raw spring constant bending stiffness data from the finite element numerical simulations.*

Supplementary Tables

Supplementary Table 1: The analytically calculated areal density of the tested nanocardboard plates based on the designed webbing geometry and an alumina density of 3900 kg m^{-3}. For the experimental samples that we weighed, the actual weight was always lower than this calculated weight by around 10%, therefore suggesting that the density of our atomic-layer-deposited alumina is lower than assumed or some of the alumina was etched during the release.

**Areal Density (g m^{-2})**		**Height (μm)**		
		**3**	**10**	**50**
**Thickness (nm)**	**50**	0.375307	0.441856	0.822133
	**100**	0.750615	0.883712	1.644266
	**400**	3.00246	3.534848	6.577064

Supplementary Table 2: Comparisons of the bending stiffnesses of nanocardboard with different webbing patterns.

	Hexagonal kagome	Triangular motif	Randomly-oriented square basketweave
Basketweave	$R = \frac{D_{\mathrm{bw}}}{D_{\mathrm{hex}}} = 1.975$	$R = \frac{D_{\mathrm{bw}}}{D_{\mathrm{tri}}} = 1.65$	$R = \frac{D_{\mathrm{bw}}}{D_{\mathrm{ran}}} = 1.76$

Supplementary Table 3: Bending stiffness and shear modulus obtained by fitting the experimental curves for the apparent bending stiffness. The values are normalized by the cantilever width. The error is provided by 95% confidence interval.

Thickness (nm)	Height (μm)	Avg. True Bending Stiffness, D_{xx} (Nm)	Error (Nm)	Avg. Shear Modulus, G (N m^{-2})	Error (N m^{-2})
100	50	4.677E-06	+/- 4.55E-07	1.19E+04	+/- 9.42E02
100	10	1.598E-07	+/- 2.32E-08	3.60E+03	+/- 8.835E02
400	50	1.221E-05	+/- 1.16E-06	6.07E+04	+/- 8.49E03
400	10	1.146E-06	+/- 1.72E-07	1.40E+05	+/- 3.38E04
50	50	1.484E-06	+/- 5.35E-07	1.66E+03	+/- 2.845E02
50	10	2.303E-08	+/- 1.05E-08	2.97E+03	+/- 1.514E03
400	3	1.922E-07	+/- 8.01E-08	1.01E+05	+/- 5.14E04
100	3	3.800E-09	+/- 3.8E-09	4.10E+05	+/- 4.100E5

Supplementary Table 4: Symbols and their SI units used in the theoretical analysis. The web is assumed to be a series of hollow plates with thickness t.

Symbol	Quantity	Dependence on t	Dependence on h	Dependence on s
EI	Bending stiffness of strands (Nm^2)	$\sim t^3$	None	None
GA	Shear stiffness of strands (N)	$\sim t$	None	None
EA	Stiffness of the strands (N)	$\sim t$	None	None
L_1	Stiffness of web along $\mathbf{e}_1$ (N/m)	$\sim t$	$\sim 1/h$	$\sim 1/s$
L_3	Stiffness of web along $\mathbf{e}_3$ (N/m)	$\sim t$	$\sim 1/h$	$\sim 1/s$
H	Bending stiffness of web (Nm)	$\sim t$	$\sim 1/h$	$\sim 1/s$

Supplementary Methods

Silicon mold fabrication

Silicon-on-insulator wafers were coated with a thin film of silicon oxide and then silicon nitride via plasma-enhanced chemical-vapor deposition to serve as the hard mask for photolithography (Oxford Plasmalab 100 PECVD). The wafers were then spin coated with hexamethyldisilizane to promote adhesion (MicroChem MCC Primer 80/20), baked at 115 °C on a hotplate for 1 min, and spin coated with a photoresist layer (Shipley Microposit S1818), and again baked at 115 °C on a hotplate for 1 min. The photomask pattern consisted of both the webbing pattern and also outlines for samples (chips) of 13-mm diameter circles. We exposed the photoresist with vacuum contact photolithography (Suss MicroTec, MA6 Gen 3, 300-500 mJ cm^{-2}), and developed in MF-319 (Shipley Microposit) for 1-1.5 min, followed by rinse in deionized water and drying under N_2 flow. The wafers were then heated on a hotplate at 115 °C for 1 min to harden the resist layer.

In order to transfer the pattern in the photoresist, we performed sequential reactive ion etching processes. The webbing and outline pattern was transferred to the silica/nitride hard mask through CHF_3/O_2 reactive ion etching (RIE) (Oxford 80 Plus) until all of the silica was removed via inspection with an optical microscope and spectral reflectometer. An RIE of CF_4 was used to remove the final oxide and etch slightly into the silicon. The pattern was then transferred into the silicon via deep reactive ion etching (SPTS DRIE) of SF_6 and C_4F_8. The time, number of cycles, and power was calibrated such that the silicon device layer was completely etched through to the buried oxide in a vertical fashion.

Silicon Mold Removal from Handle Wafer

To remove the samples/chips from the wafer, we immersed the wafer upside down in a bath of 49% hydrofluoric acid for >1 hour to etch the oxide hard mask and buried oxide layer. After carefully rinsing with deionized water, a few of the chips self-released from the wafer, though most of the chips remained stuck. To remove the rest of the chips, we slowly inserted a razor blade between the chip and the handle wafer inside of a water bath, lifting the chip from the wafer. Finally, all of the released chips were air dried.

Atomic Layer Deposition

The structural alumina was deposited with atomic layer deposition on individual chips (Cambridge Nanotech S200 ALD). Chips were taped to a custom glass carrier such that both the top and bottom sides were exposed for deposition. The deposition process was carried out at 250 °C with a pulse of H_2O vapor for 0.015 sec, delay of 5 sec, pulse of tetramethylaluminum for 0.015 sec, and delay of 5 sec. Each cycle deposited an incomplete layer of amorphous aluminum oxide of 0.9 to 1.0 angstrom. We measured the final deposited thickness on a separate piece of prime silicon with spectral reflectometry (Filmetrics, F40 model).

Laser Machining of Cantilevers and Mounting

ALD-coated chips were taped to a flat carrier wafer for laser micromachining into individual cantilevers of 2-12 mm length and 0.5 mm width. We used an IPG IX280-DXF green laser at 50% power and 100 kHz rep rate to define the outline of the cantilevers. Between 1 and 250 repetitions of the outline was required completely etch through the chip, depending on the silicon thickness. After etching, the cantilevers were mounted on glass slides with UV-curing epoxy (Loon Outdoors, High Viscosity). During mounting, we aimed to minimize damage to the cantilever and limit the epoxy creeping along the cantilever beyond the small section of the cantilever that contacted the glass slide.

Etching of Silicon Mold

The silicon mold was etched with XeF_2 vapor (Xactics/SPTS), leaving only the external hollow nanocardboard structure. For the etching of large pieces (> 1 mm on any side), the edges of the chips were slightly fractured with a razor blade as to expose an etch surface of the inner silicon. They were mounted on a glass slide and covered with aluminum foil as to prevent the nanocardboard device from moving during etching. Full etching required >500 cycles at 60 sec cycle time and 2 T pressure. For nanocardboard cantilevers, the mounted slides were covered with aluminum foil, though etching required fewer cycles as more surface area of the silicon was exposed and less total silicon etching was required. The etching was completed when the nanocardboard became optically translucent and the dark silicon region had clearly disappeared.

Atomic Force Microscopy Characterization

In order to characterize the spring constant of fabricated cantilevers, we made use of an atomic force microscope (AFM) (Asylum MFP-3D) at room temperature and calibrated, commercial AFM probes. In order to determine the spring constant of the AFM probes, we followed the Sader method integrated into the IGOR software (Version 6.37). For each set of data (ie., a displacement curve at a particular length along the nanocardboard cantilever), we determined where the base of the nanocardboard cantilever was through an optical microscope, and then took measurements (by measuring the reaction displacement of the AFM probe as it moved through a z-displacement of 10 μm) every 5 unit cells towards the tip by moving the stage with micrometers. AFM displacement measurements were taken at a speed of 1 or 2 sec per measurement. In order to extract the cleanest measurement, many displacements were required per location before we converged on the expected "hockey stick" shaped curve. When possible, we took measurements at more than one point along the width of the cantilever, and averaged the respective spring constant values. For each of the data points presented in Fig. 2 of the main text, only a single cantilever was tested, but Supplementary Fig. 4 presents the bending testing of many dozen different cantilevers at only the tip to show that the characteristics are consistent and reproducible. We exported the raw z-stage position and calculated nanocardboard deflection data for each measurement with post-processing.

To calculate the spring constant, we plotted the force displacement graph (raw z-position vs. nanocardboard cantilever force as calculated by the AFM probe calibration) and chose the contact portion of the curve to be fitted with a linear line. Typically, this chosen portion was the first 300 nm of AFM head displacement, as 300 nm was the limit of our simulation capabilities for small deflection. In some cases, the noise in the data was too large to obtain representative data from only the first 300 nm. In these cases, many micrometers of displacement were chosen for the fitting lines as to average out the noise. The slope of the fitting line was the spring constant of the combined nanocardboard cantilever and AFM probe, k_{total}, which was then used calculate the individual nanocardboard cantilever spring constant, k_{cant}, as $1/k_{total} = 1/k_{probe} + 1/k_{cant}$. The value of k_{cant} was then used to calculate the apparent bending stiffness D_{app} of that cantilever at the respective location. The apparent bending stiffness values used in this report were normalized by

the width of the cantilevers, which was nominally 0.5 mm, but measured directly with optical microscopy for each sample.

Weight measurements

We weighed a selection of nanocardboard chips on a Perkin Elmer AD4/C655-0001 system. Samples were loaded onto the scale immediately after XeF_2 etching of the silicon mold. The readout resolution was 0.1 microgram, a precision of 0.2 micrograms, and accuracy of +/- 6 micrograms. For comparison, our tested samples weighed roughly 50-100 micrograms.

Testing in Liquids

Few samples were introduced to liquid environments after etching of the silicon mold. Samples were inserted into puddles of pure deionized water or acetone for up to 5 min. In many cases, the samples would not sink under their own weight and required manual force in order to fully submerge. The liquid puddle was allowed to naturally evaporate under ambient conditions and then the evaporation of the liquid from the inter-face plate region was observed under optical microscopy.

Microscopy

Optical microscopy was performed with Zeiss Smartzoom5 2D/3D Optical Microscope and Zeiss Imager-M2m Optical Microscope under ambient conditions. Scanning electron microscopy (SEM) was performed on three different instruments. 1) JEOL 7500F High-Resolution Scanning Electron Microscope, accelerating voltage 5-15 kV, working distance 6-15 mm, in-lens detector. 2) FEI Quanta 600 Environmental Scanning Electron Microscope, accelerating voltage 5-20 kV, with and without water vapor. 3) FEI Strata DB235 Focused Ion Beam, accelerating voltage 5-15 kV, working distance 10 mm, SE detector.

Supplementary Note 1: Face sheet wrinkling and perforation pattern

The wrinkling or buckling of face sheets in sandwich structures, a well-known occurrence in macroscopic sandwich plates,[1] can limit the predictability of the mechanical properties and the overall stiffness. For some designs of the nanocardboard, in which the webbing was a hexagonally-periodic array of cylinders (Supplementary Fig. 2a), we observed the wrinkling of the face sheet for thicknesses of 100 nm or less (Supplementary Fig. 2b). The wrinkles formed over entire cantilevers because the webbing cylinders were disconnected (not forming a continuous array like in honeycomb cores), and furthermore were widely spaced. Supplementary Figure 2c provides a diagram showing how a straight line can be drawn through the face sheet without intersecting any webbing cylinders. The face sheets can therefore buckle or wrinkle along these straight lines. Accordingly, the wrinkling was experimentally observed along the 0°, 60° and 120° directions of the hexagonal lattice (Supplementary Fig. 2d). During mechanical characterization with an atomic force microscope probe, we observed irreproducible stress-strain curves which were highly nonlinear. The wrinkles moved and reoriented during each test, resulting in inconsistent mechanical responses.

In order to satisfy the "no-straight-line rule" and prevent wrinkling, we replaced the hexagonal cylinder motif with a simple basketweave motif for all subsequent experimental samples and numerical simulations. Interestingly, in our previous corrugated plates,[2] we used a similar no-straight-line rule to increase the stiffness of corrugated single-layer plates. However, in nanocardboard, the no-straight-line rule does not necessarily increase the bending stiffness. Instead, its main purpose is to prevent the formation of wrinkles along straight lines that pass between the webbing cylinders and therefore produce plates with consistent and predictable mechanical properties. Supplementary Figure 2e shows how a straight line is not able to be drawn through a basketweave face sheet without being interrupted by the webbing rectangles, thus the design eliminated wrinkles.

There are other possible webbing patterns that one could formulate for the nanocardboard structure. The general requirements for the pattern include satisfying the no-straight-line rule, ensuring that the face sheet is continuous (or the perforations must not connect with one another), and the perforations must be as small as possible (generally ensuring high stiffness of the face

sheet). Recently, researchers have reported on the mechanical properties of thins sheets with high aspect-ratio periodic perforations, specifically investigating conditions with negative Poisson's ratio. The square basketweave pattern and the hexagonal kagome pattern are the two most studied patterns for their nearly-isotropic controllable elastic modulus and Poisson's ratio.[3–6] Supplementary Table 2 and Supplementary Fig. 3 compare the simulated bending performance of basketweave nanocarboard cantilevers with a set of other perforation/webbing patterns based on that work. (Discussion of the finite-element simulations is included in Supplementary Note 3.) The simulated true bending stiffnesses, for thicknesses t of 100 nm and height h of 10 μm, are: hexagonal kagome pattern with an angle of 60 degrees, 8.2×10^{-8} N m (the simulated bending stiffness for the 52 degrees condition[4] was comparable), the analogous triangle motif, 9.8×10^{-8} N m, and randomly-angled basketweave (identical to ref. [5]) 9.2×10^{-8} N m. All of these stiffnesses are similar, yet smaller than the measured true bending stiffness of the comparable basketweave-pattern nanocardboard samples.

Supplementary Note 2: Supplement bending stiffness data and discussion

While we successfully fabricated nanocardboard cantilevers and larger pieces with an alumina thickness of 25 nm, the bending stiffness for this thickness was not fully characterized due to lower repeatability and increased measurement noise; however, a few data points are provided in Supplementary Fig. 4 for reference. Samples with thicknesses of roughly 50, 100 and 400 nm and heights of roughly 3, 10 and 50 μm were fully characterized with AFM bending measurements.

In addition to the bending stiffness (D_{xx}) calculated from the bending tests performed along the length of cantilevers, we also measured the apparent bending stiffness of many samples solely at the tips of cantilevers. While this data was not used to calculate the reported D_{xx} and G, it does follow similar trends to the "along the length" data shown in Fig. 2 of the main text and matched well with numerical simulations. Supplementary Figure 4 shows a plot of all of these such data points, the apparent bending stiffness as a function of the length of the cantilever at the tip, to complement the discussion in the main text. There is significant variability (on the order of 10%) from sample to sample, representative of the experimental error and noise naturally present during the testing of >50 cantilevers of different parameters. Each condition follows the expected trend of increasing stiffness with length. The solid horizontal lines designate the theoretically expected and simulated saturated bending stiffness of each experimental condition.

The data in Supplementary Table 3 shows that the nanocardboard true bending stiffness scales as expected over an order of magnitude with both thickness and height. More specifically for D_{xx} (Fig. 2c in the main text), the simulation fitting-determined data points matched the theoretical values within 19% relative error, suggesting that our numerical model provides an accurate prediction of the bending stiffness. Similarly, the experimental fitting-determined D_{xx} data points match the theoretical trends (relative error < 38%) at large t, large D_{app} and high L_{90} ($t \geq 100\ \mathrm{nm}$, $h/t \geq 100$). There are three major cases that incurred significant error in which the deviations are > 50%. Firstly, for t = 50 nm it was difficult to maintain a low relative error in the deposited thickness of the ALD alumina. The second deviation came from increased experimental noise during the AFM testing of low D_{app} samples compared to relatively noise-free cantilevers with larger D_{app} (see Supplementary Fig. 5a and 4b for example force-displacement curves). Lastly, nanocardboard cantilevers with low h/t, or low L_{90} , had relatively fewer unsaturated D_{app}

data points available for accurate fitting. As for G (Fig. 2d in the main text), the simulation fitting-determined data points match the theoretical trends within 40% relative error except for: the nanocardboard cantilevers with 400 nm thickness, again where the fitting was error-prone due to the relatively few data points in the low L, shear-dominated region. The experimental fitting-determined G data points only match (relative error < 32%) with our theoretical model for cantilevers with large D_{app} and high l_{90} ($h = 50\ \mu m$, $h/t \geq 500$). We attribute this discrepancy to insufficiently stiff clamping of the cantilevers at the base since the discrepancy was observed for the stiffest and shortest cantilevers and inadequate clamping is known to reduce the measured spring constant for short beams.

Supplementary Note 3: Finite element simulations of nanocardboard

Numerical simulations were carried out to validate the experimental observation of the nanocardboard subjected to out-of-plane force. The nanocardboard model was designed in AutoCAD 2014 and then exported to Abaqus v6.11-2. Supplementary Figure 7a demonstrates the initial configuration, boundary and loading conditions of the cantilevered nanocardboard. To simulate the experimental setup of the cantilevered nanocardboard, a clamped boundary condition was applied to the left end of the beam, while the location of the external loading was changed along the beam length. Shell element (S4R) was used in the finite element (FE) model. The calculation step was static without the consideration of Nlgeom. The geometric properties of the cases numerically simulated are summarized as follows: Unit length of 94 with 30 total units; cantilever width of 500 μm, cantilever height of 3, 10 and 50 μm and film thickness of 25, 50, 100 and 400 nm; Young's Modulus of 130 GPa and Poisson's ratio of 0.22; Element size of 10 μm and type of S4R.

Supplementary Figures 7b and 7c compare the deformed shape of the nanocardboard for different load locations in order to highlight shear- and bending-dominated deformation. The load location was varied as (I) 470 μm, (II) 940 μm, (III) 1410 μm, (IV) 2350 μm, (V) 3290 μm, and (VI) 3760 μm from the base of the cantilever. Supplementary Figure 7b displays the deformation configuration from a side view. The deformation is shear-dominated when the loading locations are (I) 470 μm, (II) 940 μm, and (III) 1410 μm, while deformation is bending-dominated when the loading locations are (IV) 2350 μm, (V) 3290 μm, and (VI) 3760 μm. Supplementary Figure 7c shows the corresponding nanocardboard deformation in a perspective 3D view. While these results are specific to a set of geometric conditions (500 μm width, 10 μm height, 0.05 μm thickness), the same trend can be observed for nanocardboard cantilevers of any conditions as is described in the theoretical Supplementary Note 5.

For the numerical simulation data presented in Figs. 2b-d of the main text, the cantilever was displaced with a given load of 1×10^{-12} N at different lengths, as described in the previous paragraph**.** The displacement of the cantilever was measured to extract the spring constant, and ultimately calculate the apparent bending stiffness for each particular condition. As was described in Supplementary Note 2, the curve of apparent bending stiffness vs. length was plotted and fitted.

From the fitted function, we extracted the simulated true bending stiffness (N m) and shear modulus (N m^{-2}). For many geometric conditions, we also verified the fitted bending stiffness by applying a pure bending moment in place of an out-of-plane force load, and measuring the resulting curvature of the deformed cantilever. Comparable results were obtained between the force- and moment-induced bending stiffnesses.

Supplementary Figure 7d presents the effect of the pattern angle, i.e., the angle between the repeated unit and nanocardboard length, on the deviation of simulated bending stiffness. The pattern angle was varied from 0° to 90°, and three thicknesses were investigated (100, 300, and 400 nm). Symmetric variations are obtained from all three cases. In particular, the minimum bending stiffness occurs when the pattern angle is 30° and 60°, with a relatively stiffening at 45°. The deviation of up to 15% was within the range of our experiment error, and therefore we did not attempt to validate this angle-dependent trend with fabricated samples. Further investigation, both experimental and theoretical, will better elucidate the anisotropic nature of the bending stiffness for the basketweave and other webbing patterns.

Supplementary Note 4: Sharp bending of nanocardboard cantilevers

The large-deformation bending of the nanocardboard cantilevers was carried out for select samples in order to determine the minimum radius of curvature that could be attained without apparent fracture or plastic deformation. As has been shown with other bulk mechanical metamaterials, the use of elements with nanoscale thickness can allow for much larger failure-free deformations than would be expected from similar macroscale materials.[7,8] Similarly, our nanocardboard plates were able to recover to their original position after being bent to have a radius of curvature < 100 μm, as show in Fig. 1h-k and Supplementary Fig. 8. To achieve such extreme deformations, we spatially translated the free-end of nanocardboard cantilevers with an Omniprobe tip inside an SEM, first bending the cantilever down, and then compressing the cantilever until the free end contacted the clamping substrate in order to induce beam buckling. The obtained SEM images were used to measure the radius of curvature for different conditions of cantilever height, film thickness, and distance of the Omniprobe tip from the base of the cantilever. Supplementary Movie 1 provides examples of the bending experiments. Note that while most of the bent cantilevers recovered to their initial positions, some of the cantilevers partially or completely fractured when the displacements were extreme or complex. Many cantilever were also damaged by the sharp probe, which sometimes punctured the nanocardboard.

The finite element models of sharply bent cantilever used the same two steps as the experiments: 1) bending to introduce buckling imperfection, and 2) compression. To fully investigate the large deformation effects, the geometric nonlinearity was taken into account. In order to ensure convergence of the model, displacements were placed at the free end of the cantilever. In particular, a displacement in the transverse direction of half of the cantilever width was added in the bending step, and a displacement in the longitudinal direction of the cantilever length was placed for the compression step. Supplementary Figures 8b, 8d, and 8g show images of the resulting deformation, where the colors represent the principle in-plane strains on the structure. The shapes of the deformations predicted by the FE model agree with the experiments. Supplementary Figure 8h and 8i present the deformation of a different sandwich-like structure with continuous face sheets. Comparing with the perforated structure, the large deformation-induced absolute strains of the continuous structure are approximately 5~10 times higher, which results in permanent damages to the face sheet material. In contrast, the FE model studies suggest that the maximum local strain for

the nanocardboard structure with the perforated face sheets was close to 1%, hence free from material strain failure as was the case in the experimental bending.

Supplementary Note 5: Theoretical modeling and geometric optimization

Theoretical scaling laws for the deformation characteristics of nanocardboard

We use the theory of an elastic double-stranded rod[9] to explore the effect of dimensions and elastic properties of the webbing on the macroscopic deformation of nanocardboard through a model biplate.

Sandwich Structure

In our experiments the nanocardboard consists of two parallel alumina plates that were connected to each other through a webbing of hollow rectangular columns. This geometry is akin to sandwich panel plates, except our plates were made of a brittle material and their dimensions in the nanometer to micrometer range. Sandwich panels have been extensively studied in the literature, both analytically[10] and through finite element simulations.[11,12] Finite element simulations of our nanocardboard are described in Supplementary Note 3; here we will focus on our analytical model. For the purposes of deriving scaling laws we will treat our two-dimensional nanocardboard (or bi-plates) as a one-dimensional bi-rod since they are loaded like a cantilever. Also, we are not aware of a theory of bi-plates, while there exists a well-established continuum theory for bi-rods (including large deformations)[9] which can account for complex geometries of the web. In contrast, analytical models for sandwich plates treat the webbing as a homogeneous elastic continuum even though in practical applications the web could be granular, like a foam.[13,14]

Our idealization of the bi-plate consists of two thin outer face sheets connected by a web as in Supplementary Fig. 9. The web itself consists of two parallel sheets of thickness t (denoted as t_{cant} in the following section) with separation h (denoted as h_{cant} in the following section) since the web in our nanocardboard is hollow. We need to estimate the transverse shear stiffness and flexural stiffness of the structure and examine the effect of parameters t, h and s (the spacing between webs) on it. This exercise has been performed for corrugated plates with a Z-core and for foam filled sandwich plates that are of central importance from the standpoint of structural engineering. Various approaches based upon the arguments from the strength of materials have been used to obtain these estimates.[10,11,13,14] In such structures, the core and the outer face sheets consist of the same thickness and the bending stiffness of both the members scale as $\sim t^3$. However, in our bi-plate $t << d << h \approx s$, as such the bending stiffness of the core $\sim th^2$ and that of outer plates $\sim t^3$.

The fact that $s >> d >> t$ and a granular web (with zero core stiffness at some places and finite at others) imparts a distinctive staircase structure to the resultant displacement profiles as shown in Fig. 2a. A homogenized modulus for the core cannot capture this. Moreover, for plane strain loading we can describe the resultant behavior using the theory of a double-stranded elastic rod.[9] The principal advantage of this theory is that we can account for the geometry of the hollow web including its granularity exactly.

Equivalence between a bi-rod and a bi-plate

Our primary concern in this section is to show that we can apply a one-dimensional (beam) theory to study the deformation of plates away from the free edges if the dimension along the transverse direction W (denoted as W_{cant} in later sections) is much larger than the thickness of the plate t. We follow ref [15]. The axial stress σ_{zz} in the bulk of a beam or a plate is directly proportional to the distance from the neutral axis or neutral plane, so that

$$\sigma_{zz} = \phi \frac{Mx}{EI}. \tag{1}$$

In the case of a beam, $W \sim t$ and $\varphi = 1$, while for a plate $W >> t$ and $\varphi = 1/(1-\nu^2)$ away from the edges[15] where ν is the Poisson ratio of the material. We show that in the case of a cantilever plate we recover the solutions for an Euler beam with E replaced by $E/(1-\nu^2)$. Consider a cantilever plate in the bottom of Supplementary Fig. 8 which is bent by the force $p_x\mathbf{e}_1 - p_z\mathbf{e}_3$ and moment $m_y\mathbf{e}_2$ at the edge $z = L$. Let the displacement of the neutral plane be $w(y, z)$. Furthermore, we assume that the plate is inextensible. For small deformations $|w| << 1$, the elastic energy can be obtained using the Kirchoff's theory of plates.

$$E = \int_s ds \int_y dy \, \frac{D}{2}\left[\left(w_{zz} + w_{yy}\right)^2 + 2(1-\upsilon)\left(w_{zy}^2 - w_{zz}w_{yy}\right)\right], \text{ where } D = \frac{Et^3}{12(1-\upsilon^2)}. \tag{2}$$

The work done by the external forces and moments at $z = L$ is given by

$$W = \int_y dy \, [m_y w_z + p_z w - p_z w w_z]|_{z=L} \approx \int_y dy \, [m_y w_z + p_x w]|_{z=L}. \tag{3}$$

If the transverse dimension of the plate $W >> t$, then away from edges $w(z, y) = w(z)$. Due to anticlastic curvature, this assumption breaks down close to the edges. We substitute this into Supplementary Equation 2 to get

$$E = \int_z dz \int_y dy \, D w_{zz}^2. \tag{4}$$

The potential energy is the difference of the elastic energy and external work done, hence

$$\text{PE} = E - W_{\text{ext}} = \int_y dy \int_z dz \, D w_{zz}^2 - \int_y dy \, \left[m_y w_z + p_x w\right]|_{z=L}. \tag{5}$$

$$\delta \text{PE} = \int_y dy \int_z dz\, D\omega_{zz}\delta w_{zz} - \int_y dy\, \left[m_y \delta w_z(L) + p_x \delta w(L)\right], \tag{6}$$

$$\delta w = \delta w_z = 0 \quad at\; z = L.$$

Setting $\delta PE = 0$, we obtain

$$D\frac{d^4 w}{dz^4} = 0, \quad z \in [0, L], \tag{7}$$

$$\text{and } Dw_{zzz} = p_x, \quad Dw_{zz} = m_y, \quad \text{at } z = L.$$

Another set of boundary conditions is $w = w_z = 0$ at $z = 0$. Thus, we recover the governing equation for the bending of an Euler-Bernoulli beam with E replaced by $E/(1-\nu^2)$ which is valid at constant loading sufficiently far from the free edges. Since the bi-plate shown in Supplementary Fig. 8 is an assembly of three such beams with $W >> t$, and the load is constant in the transverse direction in our experiments, the equations of a bi-rod should accurately describe the mechanics of a bi-plate away from the free edges.

Assumptions of the bi-rod model

The geometry of the problem is presented in Supplementary Fig. 8. The bi-rod consists of:

- Outer Strands: The outer strands denoted by ± are elastic rods capable of undergoing bending, extension and shear deformation. Any quantity, say **a**, pertaining to ± strands is denoted by $\mathbf{a}^{\pm}$. For instance, displacement of the upper strand in the direction of $\mathbf{e}^1$ is u_1^+. The two strands have identical elastic properties.
- Inner Web: The outer strands are connected to each other via an elastic web which is capable of transferring both forces and moments. The web is capable of undergoing extension, shear and bending.

Kinematics

We consider a general planar deformation in $\mathbf{e}_1$, $\mathbf{e}_2$ plane and specialize the equations of Moakher and Maddocks.[9] For the ± strand the kinematic variables $u_1^{\pm}$ and $u_3^{\pm}$ denote the displacements in $\mathbf{e}_1$ and $\mathbf{e}_3$ directions, respectively and $\theta^{\pm}$ denotes the orientation of the cross-section.

$$\mathbf{r}^+ = (z + u_3^+)\mathbf{e}_3 + u_1^+\mathbf{e}_1, \tag{8a}$$

$$\mathbf{r}^- = (z + u_3^-)\mathbf{e}_3 + u_1^-\mathbf{e}_1. \tag{8b}$$

$\mathbf{r}^+$ and $\mathbf{r}^-$ are position vectors of a point located at z.

Governing Equations

We assume $\boldsymbol{n}^{+}$ and $\boldsymbol{n}^{-}$ are the internal force vectors in the outer strands and $\boldsymbol{m}^{+}$ and $\boldsymbol{m}^{-}$ are the internal moments in the outer strands, then

$$\boldsymbol{n}_z^{+} - \boldsymbol{f} = 0 \tag{9a}$$

$$\boldsymbol{n}_z^{-} + \boldsymbol{f} = 0 \tag{9b}$$

$$\boldsymbol{m}_z^{+} + \mathbf{r}_z^{+} \times \boldsymbol{n}^{+} + \frac{1}{2}(\mathbf{r}^{+} - \mathbf{r}^{-}) \times \boldsymbol{f} - \boldsymbol{c} = 0 \tag{10a}$$

$$\boldsymbol{m}_z^{-} + \mathbf{r}_z^{-} \times \boldsymbol{n}^{-} + \frac{1}{2}(\mathbf{r}^{+} - \mathbf{r}^{-}) \times \boldsymbol{f} + \boldsymbol{c} = 0. \tag{10b}$$

Here $\boldsymbol{f}$ and $\boldsymbol{c}$ are the force and moment exerted by + strand on − strand. These are related to the deformations of the web via elastic constitutive laws.

Boundary Value Problem

We substitute the elastic constitutive relations for the outer strands and the web in the governing equations to get:

- For the + rod:

$$GA(u_{1z}^{+} - \theta_S^{+}) - L_1(u_1^{+} - u_1^{-}) = 0 \tag{11a}$$

$$EAu_{3zz}^{+} - L_3[(u_3^{+} - u_3^{-}) + a(\theta^{+} - \theta^{-})] = 0 \tag{11b}$$

$$EI\theta_{zz}^{+} + GA(u_{1z}^{+} - \theta^{+}) - aL_3[(u_3^{+} - u_3^{-}) + a(\theta^{+} - \theta^{-})] - H(\theta^{+} - \theta^{-}) = 0 \tag{11c}$$

- For the − rod:

$$GA(u_{1zz}^{-} - \theta_S^{+}) - L_1(u_1^{+} - u_1^{-}) = 0 \tag{12a}$$

$$EAu_{3zz}^{-} - L_3[(u_3^{+} - u_3^{-}) + a(\theta^{+} + \theta^{-})] = 0 \tag{12b}$$

$$EI\theta_{zz}^{-} + GA(u_{1z}^{-} - \theta^{-}) - aL_3[(u_3^{+} - u_3^{-}) + a(\theta^{+} + \theta^{-})] + H(\theta^{+} - \theta^{-}) = 0 \tag{12c}$$

Notice

$$\boldsymbol{f} = L_1(u_1^{+} - u_1^{-})\mathbf{e}_1 + L_3[(u_3^{+} - u_3^{-}) + a(\theta^{+} + \theta^{-})]\mathbf{e}_3 \tag{13}$$

and

$$\boldsymbol{c} = H(\theta^{+} - \theta^{-})\boldsymbol{e}_2 \tag{14}$$

Boundary conditions are:

- $z = 0$

$$u_1^{\pm} = u_3^{\pm} = \theta^{\pm} = 0 \tag{15}$$

- $z = L$

$$u_{1z}^{+} - \theta^{+} = \frac{P}{GA} \quad u_{1z}^{-} - \theta^{-} = \frac{P}{GA} \quad u_{3z}^{\pm} = 0 \quad \theta_z^{\pm} = 0 \tag{16}$$

Adding and subtracting the governing equations given above and setting $u_1^+ + u_1^- = u_i$, $u_1^+ - u_1^- = u_i^c$ for $i = 1, 3$, $\theta^+ + \theta^- = \theta$ and $\theta^+ - \theta^- = \theta^c$

$$u_{1z} - \theta_z = 0 \tag{17a}$$

$$u_{3zz} = 0 \tag{17b}$$

$$EI\theta_{zz} + GA(u_{1z} - \theta) - 2aL_3(u_3^c + a\theta) = 0 \tag{17c}$$

$$u_{1zz}^c - \theta_z^c - \frac{2L_1}{GA}u_1^c = 0 \tag{17d}$$

$$u_{3zz}^c - \frac{2L_3}{EA}(u_3^c + a\theta) = 0 \tag{17e}$$

$$EI\theta_{zz}^c + GA(u_{1z}^c - \theta^c) - 2H\theta^c = 0 \tag{17f}$$

Boundary conditions can be similarly obtained.

- $z = 0$

$$u_1 = u_1^c = u_3 = u_3^c = \theta = \theta^c = 0 \tag{18}$$

- $z = L$

$$\theta_z = \theta_z^c = u_{3z} = u_{3z}^c = 0, \qquad u_{1z} - \theta = u_{1z}^c - \theta^c = \frac{P}{GA} \tag{19}$$

The governing Supplementary Equations 10 and boundary conditions Supplementary Equations 11 are solved using MATLAB to get the transverse displacement u_1 $(z = L) = \delta$ (say) at $z = L$ which is then used to calculate the effective bending stiffness using

$$K_\mathrm{b} = \frac{F}{\delta}\frac{L^3}{3}. \tag{20}$$

Results

In order to respect the granular nature of the web, we assume that the elasticity of the web is a function of the arclength parameter.

$$L_1(z) = L_1^0 f(z) \quad L_3(z) = L_3^0 f(z) \quad H(z) = H^0 f(z) \tag{21}$$

Here $f(z)$ is a modulating function with period s. For convenience, we choose

$$f(z) = e^{-50s\quad ^2(\pi z/s)}. \tag{22}$$

We then solve the system of ordinary differential equations of the previous section to find the deformation of the bi-rod in response to loading of $F = 5$ µN as in a cantilever of $W = 1$ m. The resultant displacement profiles exhibit a distinctive staircase character as shown in Fig. 2a.

The staircase-like displacement profile of the end-loaded cantilever is a result of the granularity of the web which a sandwich plate model with a homogeneous web cannot capture. From our experiments and finite element calculations we know that the deformation of our nanocardboard could be shear-dominated or bending-dominated depending on the geometry of the specimens. For a Timoshenko beam, the displacement at the end z = L contains contributions both from shear and bending, so that

$$u(z = L) = \frac{FL^3}{3EI} + \frac{PL}{GA}. \tag{23}$$

In the above, EI is the bending stiffness of the Timoshenko beam and GA is its shear stiffness. For such a system, we say that the effective bending stiffness is K_b which is calculated as follows:

$$\frac{FL^3}{3E} + \frac{FL}{GA} = \frac{FL^3}{3K_b}$$

$$\frac{K_b(L)}{EI} = \frac{1}{1+\frac{3EI}{GAL^2}} = \frac{L^2}{L^2+c_0} \quad \text{where } c_0 = \frac{3EI}{GA} \tag{24}$$

$$\frac{K_b(L)}{EI} \sim \begin{cases} L^2 & \text{if } L \ll \sqrt{c_0} \\ 1 & \text{if } L \gg \sqrt{c_0} \end{cases}$$

As the length of the plate keeps increasing the effective bending stiffness increases and eventually saturates. L_{90} is the length of the plate at which $K_b = 0.9EI$.

$$L_{90} = \sqrt{\frac{c_0}{1/f-1}} = \sqrt{\frac{3EI/GA}{1/f-1}}, \qquad f = 0.9. \tag{25}$$

In order to calculate L_{90} for a bi-plate, we need the effective bending stiffness EI_{eff} and effective shear stiffness GA_{eff} which replace EI and GA, respectively in the above equation. We can plot u(z = L) vs. length of rod L and fit a cubic polynomial through the points and extract the effective bending stiffness EI_{eff} and effect shear stiffness GA_{eff} from the fit.

$$u_L = u(z = L) = a_3L^3 + a_2L^2 + a_1L + a_0. \tag{26}$$

From the fit, we find that $a_2 \approx a_0 \approx 0$.

$$u_L = u(z = L) = a_3L^3 + a_1L$$

$$\text{where} \quad a_3 = \frac{F}{3EI_{\text{eff}}} \quad a_1 = \frac{F}{GA_{\text{eff}}}$$

$$\text{Effective Bending Stiffness:} \quad EI_{\text{eff}} = \frac{F}{3a_3} \tag{27}$$

$$\text{Effective Shear Stiffness:} \quad GA_{\text{eff}} = \frac{F}{a_1} \tag{28}$$

$$L_{90} = \sqrt{\frac{3EI_{\text{eff}}/GA_{\text{eff}}}{1/f-1}} = \sqrt{\frac{a_1/a_3}{1/f-1}} \quad f = 0.9 \tag{29}$$

Note that a_3 and a_1 are proportional to P which makes EI_{eff} and GA_{eff} independent of P. In case of a homogeneous web, we compared our result for L_{90} using the above methodology with the numerical result obtained by integrating the ordinary differential equations with various lengths L of the bi-rod, computing their K_b using Supplementary Equation 24, and then getting L_{90} from a plot of K_b vs. L. We found that L_{90} computed using both methods agreed very well. Thus, we apply the method based on fitting with a cubic polynomial to compute L_{90} for a bi-rod with a granular web.

Variation with the thickness *t*, height *h*, and period *s*

We obtained the results in Supplementary Fig. 10 for variation of effective bending stiffness EI_{eff}, effective shear stiffness GA_{eff} with the thickness t of the plate, height h of the cantilever and period s between the webbing structures. The scaling of each stiffness with various geometrical parameters of our bi-rod are summarized in Supplementary Table 4. Note that while the absolute values of the apparent bending stiffness and shear stiffness are reasonably close to the values for the nanocardboard cantilevers in the experiments, we do not expect an exact match since the theoretical bi-rod presented in this section is not identical to the basketweave pattern in the experiments.

With the above information taken into account, we found the following scaling relationships: $L_{90} \sim {}^{hs}/_{t}$, $EI_{\text{eff}} \sim th^2$, $GA_{\text{eff}} \sim {}^{t^3}/_{s^2}$. These scaling relationships are graphed and represented in Fig. 2 of the main text. Note also that $L_{90} \propto \sqrt{\frac{EI_{\text{eff}}}{GA_{\text{eff}}}}$.

Optimal Design of the Cantilever

Problem Statement

For cantilevers and plate structures, designing for the minimum deflection or maximum resistance to deflection is often desirable in a wide variety of situations, including transportation, construction materials, and biological skeletons or shells. Therefore, the optimization in this section seeks to maximize the spring constant of the cantilever k_{cant} for any chosen areal density AD_{cant}. Since

the shear component of deflection tends to dominate for cantilevers on the order of a few millimeters, the optimization is not as simple as maximizing the bending stiffness D_{xx}. In the following, we analytically design the stiffest and lightest nanocardboard cantilever considering both bending and shear deformations. The optimization focuses on only the basketweave patterns that can be reliably fabricated and do not exhibit spontaneous wrinkling, but the derivation also provides insight into the maximum achievable enhancement factor of the structure and how the optimal designs scale for different geometric or density constraints.

Two design variables of the presented cantilever are particularly investigated, i.e., the cantilever height h_{cant}, and the rectangle length l_{rect} of the basketweave pattern. Increasing the bending stiffness requires increasing the cantilever height, which also increases the mass of the cantilever. Increasing the shear stiffness, however, requires decreasing the rectangle length of the basketweave pattern, which also increases the mass of the cantilever. Therefore, for a fixed mass or areal density, there is a tradeoff between increasing the height to reduce the bending displacement and decreasing the rectangle length to reduce the shear displacement. Mathematically, the optimization can be described as

$$\begin{cases} \mathbf{Max}[k_{\text{cant}}(h_{\text{cant}},\ l_{\text{rect}})] \\ \text{Subject to:}\quad \text{AD}_{\text{cant}}(h_{\text{cant}},\ l_{\text{rect}}) = \Gamma \end{cases}, \tag{30}$$

where Γ is an arbitrary value of the areal density of the cantilever.

The Lagrange multiplier method is used to optimize the cantilever. The Lagrange expression is defined as

$$\mathbf{L}(h_{\text{cant}},\ l_{\text{rect}},\ \lambda) = k_{\text{cant}} - \lambda \text{AD}_{\text{cant}}\ , \tag{31}$$

where λ represents the Lagrange multiplier. Therefore, the optimization in Supplementary Equation 30 is rewritten as

$$\begin{cases} \nabla_{h_{\text{cant}},\ l_{\text{rect}}} \mathbf{L}(h_{\text{cant}},\ l_{\text{rect}}) = \left(\frac{\partial \mathbf{L}}{\partial h_{\text{cant}}}, \frac{\partial \mathbf{L}}{\partial l_{\text{rect}}}\right) = 0 \\ \text{AD}_{\text{cant}} = \Gamma \end{cases}. \tag{32}$$

To derive the optimal h_{cant} and l_{rect}, we need to use the expressions for the spring constant and areal density of the cantilever, described in the following sections.

Spring Constant and Areal Density of the Cantilever

Spring Constant

Following Timoshenko beam theory, the deflection δ_{cant} of the cantilever consists of two components, i.e., bending-induced and shear-induced deflections, which are given as

$$\begin{cases} \delta_{\text{bending}} = \frac{20FL_{\text{cant}}^3}{9Et_{\text{cant}}h_{\text{cant}}{}^2W_{\text{cant}}} \\ \delta_{\text{shear}} = \frac{FL_{\text{cant}}}{Gh_{\text{cant}}W_{\text{cant}}} \end{cases}, \tag{33}$$

and the total cantilever deflection is

$$\delta_{\text{cant}} = \delta_{\text{bending}} + \delta_{\text{shear}} \,. \tag{34}$$

The spring constant of the cantilever is, therefore, given by:

$$k_{\text{cant}} = \frac{F}{\delta_{\text{cant}}} = \frac{9EGh_{\text{cant}}^2 t_{\text{cant}} W_{\text{cant}}}{20\eta L_{\text{cant}}^3 + 9Eh_{\text{cant}}L_{\text{cant}}t_{\text{cant}}}, \tag{35}$$

where E is the Young's Modulus of the solid material and the shear modulus is

$$G = \eta \frac{t_{\text{cant}}^3}{h_{\text{cant}}\left(\frac{w_{\text{rect}}}{2} + l_{\text{rect}}\right)^2}, \tag{36}$$

where η, l_{rect}, and w_{rect} refer to the shear modulus constant of the plate metamaterial (obtained empirically by fitting experimental and finite element results), webbing rectangle length and webbing rectangle width of the basketweave, respectively. To simplify the optimization, we will sometimes assume below that the rectangle width is negligible ($w_{\text{rect}} = 0$) while the gap is half of the rectangle length ($g_{\text{rect}} = \frac{l_{\text{rect}}}{2}$). These are reasonable approximations of the actual nanocardboard webbing design implemented in experiments and numerical simulations, which used finite but small rectangle width. We note that these idealized parameters still satisfy the no-straight-line rule, i.e., $g_{\text{rect}} \leq \frac{1}{2}(l_{\text{rect}} - w_{\text{rect}})$, to prevent wrinkling and give a bending stiffness D_{xx} equal to 30% that of the ideal bending stiffness D_{ideal} (see discussion in main text). Supplementary Equation 36 is, therefore, reduced to

$$G = \eta \frac{t_{\text{cant}}^3}{h_{\text{cant}} l_{\text{rect}}^2} \,. \tag{37}$$

Substituting Supplementary Equation 37 into Supplementary Equation 35, we have the spring constant of the cantilever as

$$k_{\text{cant}} = \frac{F}{\delta_{\text{cant}}} = \frac{9E\eta h_{\text{cant}}^2 t_{\text{cant}}^3 W_{\text{cant}}}{20\eta L_{\text{cant}}^3 t_{\text{cant}}^2 + 9Eh_{\text{cant}}^2 l_{\text{rect}}^2 L_{\text{cant}}} \,. \qquad 38)$$

Areal Density

The areal density $\mathrm{AD_{cant}}$ of the cantilever is given as

$$\mathrm{AD_{cant}} = \rho_s(\alpha + \beta h_{\mathrm{cant}})t_{\mathrm{cant}} \,, \tag{39}$$

where ρ_s is the density of the cantilever material. α and β, the unit areal density constants for the face sheets and core of the cantilever, respectively, are determined by the basketweave design pattern, as shown in Supplementary Fig. 1 (shaded in blue),

$$\begin{cases} \alpha = 2\frac{\left((2g_{\mathrm{rect}}+l_{\mathrm{rect}}+w_{\mathrm{rect}})^2-4l_{\mathrm{rect}}w_{\mathrm{rect}}\right)}{(2g_{\mathrm{rect}}+l_{\mathrm{rect}}+w_{\mathrm{rect}})^2} & \text{(Face sheets)} \\ \beta = \frac{8(l_{\mathrm{rect}}+w_{\mathrm{rect}})}{(2g_{\mathrm{rect}}+l_{\mathrm{rect}}+w_{\mathrm{rect}})^2} & \text{(Core)} \end{cases} . \tag{40}$$

Using the simplifications $w_{rect} = 0$ and $g_{\mathrm{rect}} = \frac{l_{\mathrm{rect}}}{2}$, Supplementary Equation 39 can be reduced to

$$\mathrm{AD_{cant}} = 2\rho_s t_{\mathrm{cant}}\left(1 + \frac{h_{\mathrm{cant}}}{l_{\mathrm{rect}}}\right) , \tag{41}$$

where

$$\begin{cases} \alpha = 2 \\ \beta = \frac{2}{l_{\mathrm{rect}}} \end{cases} . \tag{42}$$

Optimization of the Enhancement Factor for the Bending Stiffness, $\mathrm{EF_b}$

We define the enhancement factor of the bending stiffness as

$$\mathrm{EF_b} = \frac{D_{\mathrm{cant}}}{D_{\mathrm{solid}}} \tag{43}$$

for any pair of a nanocardboard cantilever and a solid rectangular beam that have the same length, width, mass, and, therefore, the same areal density, $\mathrm{AD_{cant}} = \mathrm{AD_{solid}}$. EF is similar to the bending shape factor Φ_B^e used to described the enhanced bending stiffness incurred with reforming a solid cylindrical beam into a different shape with the same cross-sectional area.[16] Note that the areal density of the rectangular solid beam can be calculated as $\mathrm{AD_{solid}} = \rho_s h_{\mathrm{solid}}$, taking into account Supplementary Equation 10, the height of the solid beam with the same areal density as a nanocardboard cantilever is

$$h_{\mathrm{solid}} = (\alpha + \beta h_{\mathrm{cant}})t_{\mathrm{cant}} \,. \tag{44}$$

Substituting Supplementary Equation 42 into Supplementary Equation 44, the equivalent height of the solid beam becomes

$$h_{\mathrm{solid}} = 2t_{\mathrm{cant}}\left(1 + \frac{h_{\mathrm{cant}}}{l_{\mathrm{rect}}}\right) . \tag{45}$$

The bending stiffness of a solid beam is

$$D_{\text{solid}} = \frac{1}{12} E h_{\text{solid}}^3 \,, \tag{46}$$

where for simplicity we neglected the Poisson ratio correction factor of up to $\frac{1}{1-\nu^2}$ for wide beams. According to the numerical calibration, we find that the bending stiffness of the nanocardboard cantilever is 30% of the ideal sandwich beam, which can be written as

$$D_{\text{cant}} = 0.3 D_{\text{ideal}} \,, \tag{47}$$

where the bending stiffness of the ideal sandwich beam is given by $D_{\text{ideal}} = \frac{1}{2} E t_{\text{cant}} h_{\text{cant}}^2$. Therefore, the bending stiffness of the nanocardboard cantilever is

$$D_{\text{cant}} = \frac{3}{20} E t_{\text{cant}} h_{\text{cant}}^2 \,. \tag{48}$$

Taking Supplementary Equation 46 and Supplementary Equation 48 into Supplementary Equation 43, we obtain the enhancement factor of bending stiffness alone as

$$\text{EF}_{\text{b}} = \frac{9}{40} \frac{h_{\text{cant}}^2 l_{\text{rect}}^3}{t_{\text{cant}}^2 (l_{\text{rect}} + h_{\text{cant}})^3} \,. \tag{49}$$

Clearly, if the aim is to maximize the bending stiffness alone, both the plate height and the rectangle length should be made as large as possible. If, however, the rectangle length is fixed due to some practical considerations, the enhancement factor can be maximized with respect to the cantilever height, yielding

$$\begin{cases} h_{\text{cant,opt}} = 2\, l_{\text{rect}} \\ \text{EF}_{\text{b,max}} = \frac{1}{30} \left(\frac{l_{\text{rect}}}{t_{\text{cant}}} \right)^2 \end{cases} , \tag{50}$$

which predicts an enhancement factor of approximately 33 333 for the rectangle length of 50 μm and thickness of 50 nm. An example plot of EF_{b} vs. AD is given in Supplementary Fig. 11a for a cantilever length *L* of 2 mm. Note that the maximum EF occurs at the peak of each thickness curve. For the actual basketweave rectangle width and gap used in experiments, the absolute maximum enhancement factor is somewhat smaller as illustrated by Fig. 3b in the main text.

In summary, the bending stiffness can in principle be increased indefinitely by increasing both the height and rectangle length. However, the resulting cantilever will become extremely soft with respect to shear displacements. Therefore, the optimization of the bending stiffness alone makes

sense only in cases where the cantilever is very long, and therefore bending dominated, and the scale of the basketweave pattern is fixed (note that the period of the basketweave pattern is equal to twice the rectangle length).

Enhancement Factor for the Spring Constant $\mathrm{EF_k}$

Similar to Supplementary Equation 43, we define the enhancement factor of spring constant as

$$\mathrm{EF_k} = \frac{k_{\mathrm{cant}}}{k_{\mathrm{solid}}}, \tag{51}$$

where k_{cant} represents the spring constant of the nanocardboard cantilever given in Supplementary Equation 38, k_{solid} refers to the spring constant of a solid beam that has the same optimal areal density as the cantilever, $\mathrm{AD_{cant}} = \mathrm{AD_{solid}}$. Given that $\mathrm{AD_{solid}} = \rho h_{\mathrm{solid}}$, the height of the solid beam with equivalent mass is

$$h_{\mathrm{solid}} = 2t_{\mathrm{cant}}\left(1 + \frac{h_{\mathrm{cant}}}{l_{\mathrm{rect}}}\right). \tag{52}$$

To compare the spring constant between the cantilever and solid beam, we assume the structures have equivalent material density ρ_{s}. Since the equivalent solid beam will be much longer than it is thick, the shear deflection can be neglected and the deflection of the solid beam subjected to tip load F is then

$$\delta_{\mathrm{solid}} = \frac{FL_{\mathrm{solid}}^3}{3EI_{\mathrm{solid}}}, \tag{53}$$

where $I_{\mathrm{solid}} = \frac{W_{\mathrm{solid}}h_{\mathrm{solid}}^3}{12}$, $W_{\mathrm{solid}} = W_{\mathrm{cant}}$ and $L_{\mathrm{solid}} = L_{\mathrm{cant}}$. Therefore, the spring constant of the equivalent solid beam is

$$k_{\mathrm{solid}} = \frac{F}{\delta_{\mathrm{solid}}} = \frac{2EW_{\mathrm{solid}}t_{\mathrm{cant}}^3}{L_{\mathrm{solid}}^3}\left(1 + \frac{h_{\mathrm{cant}}}{l_{\mathrm{rect}}}\right)^3. \tag{54}$$

In order to obtain the optimal enhancement factor $\mathrm{EF_{k,opt}}$, the spring constant of the cantilever k_{cant} is maximized with respect to the cantilever height h_{cant} and rectangle length l_{rect}. In particular, substituting Supplementary Equation 38 and 41 into Supplementary Equation 32, the optimal cantilever height $h_{\mathrm{cant,opt}}$, and rectangle length $l_{\mathrm{rect,opt}}$ can be determined as

$$\begin{cases} h_{\mathrm{cant,opt}} = \sqrt[4]{\frac{5\eta}{9E{\rho_{\mathrm{s}}}^2}}\sqrt{L_{\mathrm{cant}}(\mathrm{AD_{cant}} - 2\rho t_{\mathrm{cant}})} \\ l_{\mathrm{rect,opt}} = 2\sqrt[4]{\frac{5\eta}{9E}}\sqrt{\frac{L_{\mathrm{cant}}\rho_{\mathrm{s}}}{\mathrm{AD_{cant}} - 2\rho_{\mathrm{s}}t_{\mathrm{cant}}}}\,t_{\mathrm{cant}} \end{cases}. \tag{55}$$

Substituting Supplementary Equation 55 into Supplementary Equation 33, we find that the bending deflection of the optimal cantilever is identical to the shear deflection as

$$\delta_{\mathrm{bending,opt}}\left(h_{\mathrm{cant,opt}}, l_{\mathrm{rect,opt}}\right) = \delta_{\mathrm{shear,opt}}\left(h_{\mathrm{cant,opt}}, l_{\mathrm{rect,opt}}\right) . \tag{56}$$

Taking Supplementary Equation 55 into Supplementary Equation 38, the optimal spring constant $k_{\mathrm{cant,opt}}$ can be determined as

$$k_{\mathrm{cant,opt}} = \frac{3\sqrt{E\eta}\,t_{\mathrm{cant}}W_{\mathrm{cant}}}{8\sqrt{5}L_{\mathrm{cant}}^{2}\rho_{\mathrm{s}}}\left(\mathrm{AD}_{\mathrm{cant,opt}} - 2\rho t_{\mathrm{cant}}\right) . \tag{57}$$

Further substituting Supplementary Eqation 54 and 57 into Supplementary Equation 51, we obtain the optimal enhancement factor of spring constant as

$$\mathrm{EF}_{\mathrm{k,opt}} = \frac{3\sqrt{\eta}{\rho_{\mathrm{s}}}^{2}}{2\sqrt{5E}}\frac{L_{\mathrm{cant}}t_{\mathrm{cant}}\left(\mathrm{AD}_{\mathrm{cant,opt}} - 2\rho_{\mathrm{s}}t_{\mathrm{cant}}\right)}{\mathrm{AD}_{\mathrm{cant,opt}}^{3}} \tag{58}$$

plotted in Supplementary Fig. 11b.

To find the maximum enhancement factor of spring constant in terms of the optimal areal density, the first derivative of Supplementary Equation 58 is used to solve for $\mathrm{AD}_{\mathrm{cant,opt}}$. Taking the areal density and Supplementary Equation 55 into Supplementary Equation 58, the maximum enhancement factor is determined as

$$\mathrm{EF}_{\mathrm{k,max}} = \frac{\sqrt{\eta}}{18\sqrt{5E}}\frac{L_{\mathrm{cant}}}{t_{\mathrm{cant}}} \tag{59}$$

when

$$\mathrm{AD}_{\mathrm{cant,opt}}\Big|_{\mathrm{EF}_{\mathrm{k,max}}} = 3\rho_{\mathrm{s}}t_{\mathrm{cant}} . \tag{60}$$

Note that Supplementary Equation 58 is affected by the term $\mathrm{AD}_{\mathrm{cant,opt}} - 2\rho t_{\mathrm{cant}}$. According to Supplementary Equation 41, the term refers to the areal density of the cantilever core $\mathrm{AD}_{\mathrm{cant}}^{\mathrm{c}}$ while $2\rho t_{\mathrm{cant}}$ represents the areal density of the cantilever face sheets $\mathrm{AD}_{\mathrm{cant}}^{\mathrm{f}}$, which can be expressed as

$$\mathrm{AD}_{\mathrm{cant}}^{\mathrm{c}} = \mathrm{AD}_{\mathrm{cant}} - \mathrm{AD}_{\mathrm{cant}}^{\mathrm{f}} = \mathrm{AD}_{\mathrm{cant}} - 2\rho_{\mathrm{s}}t_{\mathrm{cant}} . \tag{61}$$

Taking Supplementary Equation 60 into Supplementary Equation 61, we have

$$\mathrm{AD}_{\mathrm{cant,opt}}^{\mathrm{c}} = \rho_{\mathrm{s}}t_{\mathrm{cant}} . \tag{62}$$

Therefore, the ratio of the face sheets areal density to the core areal density for the optimal cantilever is

$$\left.\frac{\mathrm{AD}_{\mathrm{cant}}^{\mathrm{f}}}{\mathrm{AD}_{\mathrm{cant}}^{\mathrm{c}}}\right|_{\mathrm{opt}} = 2\ . \tag{63}$$

The optimal areal density ratio in Supplementary Equation 63 is identical to the optimal weight ratio of the face weight to the core weight for the optimal web-core sandwich structures.[17] This is because the rectangle width of the cantilever is assumed zero, i.e., $w_{\mathrm{rect}} = 0$, which results in the face areal density equivalent to the areal density of the web-core sandwich structures. However, the bending stiffness of the cantilever is still 30% of the ideal sandwich beam (as discussed in Supplementary Equation 47), given the cuts of the basketweave pattern on the face sheets.

The maximum enhancement factor of the spring constant is

$$\mathrm{EF}_{\mathrm{k,max}} \approx 0.0982\frac{L_{\mathrm{cant}}}{t_{\mathrm{cant}}} \tag{64}$$

for our alumina cantilevers with the geometric and material properties summarized below. Substituting the values into Supplementary Equation 55, the maximum optimal cantilever height and rectangle length are

$$\begin{cases} h_{\mathrm{cant,max}} \approx 1.716\sqrt{L_{\mathrm{cant}}t_{\mathrm{cant}}} \\ l_{\mathrm{rect,max}} \approx 3.432\sqrt{L_{\mathrm{cant}}t_{\mathrm{cant}}} \end{cases}. \tag{65}$$

Geometric and material properties of the analytically modeled cantilever

L_{cant}: 2-10 mm

t_{cant}: 50 nm

w_{rect}: 0 μm

g_{rect}: $\frac{l_{\mathrm{rect}}}{2}$

W_{cant}: 500 μm

η: 2030 GPa

E: 130 GPa

ρ_s: 3900 kg m^{-3}

Nomenclature

		Cantilever	Solid beam	Ideal sandwich
Full-size beam	Length	L_{cant}	L_{solid}	L_{ideal}
	Height	h_{cant}	h_{solid}	h_{ideal}
	Thickness	t_{cant}	t_{solid}	t_{ideal}
	Width	W_{cant}	W_{solid}	W_{ideal}
Rectangles in face sheets	Length	l_{rect}	--	--
	Width	w_{rect}	--	--
	Gap	g_{rect}	--	--
Material properties	Young's modulus	E		
	Shear modulus	G		
	Solid volumetric density	ρ_s		
	Poisson's ratio	v		
Mechanical properties	Areal density	AD_{cant}	AD_{solid}	AD_{ideal}
	Bending stiffness (a.k.a. flexural modulus)	D_{cant}	D_{solid}	D_{ideal}
	Apparent bending stiffness	D_{app}	--	--
	True bending stiffness along the length of cantilever	D_{xx}	--	--
	Spring constant	k_{cant}	k_{solid}	k_{ideal}
Comparison	Enhancement factor for bending stiffness	EF_{b}		
	Enhancement factor for spring constant	EF_{k}		

Supplementary References

1. Bitzer, T. N. *Honeycomb Technology: Materials, Design, Manufacturing, Applications and Testing*. (Springer Science & Business Media, 2012).
2. Davami, K. *et al.* Ultralight shape-recovering plate mechanical metamaterials. *Nat. Commun.* **6,** 10019 (2015).
3. Carta, G., Brun, M. & Baldi, A. Design of a porous material with isotropic negative Poisson's ratio. *Mech. Mater.* **97,** 67–75 (2016).
4. Carta, G., Cabras, L. & Brun, M. Continuous and discrete microstructured materials with null Poisson's ratio. *J. Eur. Ceram. Soc.* **36,** 2183–2192 (2016).
5. Grima, J. N., Mizzi, L., Azzopardi, K. M. & Gatt, R. Auxetic Perforated Mechanical Metamaterials with Randomly Oriented Cuts. *Adv. Mater.* **28,** 385–389 (2016).
6. Shan, S., Kang, S. H., Zhao, Z., Fang, L. & Bertoldi, K. Design of planar isotropic negative Poisson's ratio structures. *Extreme Mech. Lett.* **4,** 96–102 (2015).
7. Kooistra, G. W. & Wadley, H. N. G. Lattice truss structures from expanded metal sheet. *Mater. Des.* **28,** 507–514 (2007).
8. Kooistra, G. W., Deshpande, V. & Wadley, H. N. G. Hierarchical Corrugated Core Sandwich Panel Concepts. *J. Appl. Mech.* **74,** 259–268 (2005).
9. Moakher, M. & Maddocks, J. H. A Double-Strand Elastic Rod Theory. *Arch. Ration. Mech. Anal.* **177,** 53–91 (2005).
10. Altenbach, H. An alternative determination of transverse shear stiffnesses for sandwich and laminated plates. *Int. J. Solids Struct.* **37,** 3503–3520 (2000).
11. Nordstrand, T., Carlsson, L. A. & Allen, H. G. Transverse shear stiffness of structural core sandwich. *Compos. Struct.* **27,** 317–329 (1994).

12. Nordstrand, T. M. & Carlsson, L. A. Evaluation of transverse shear stiffness of structural core sandwich plates. *Compos. Struct.* **37,** 145–153 (1997).

13. Fung T. C. & Tan K. H. Shear Stiffness for Z-Core Sandwich Panels. *J. Struct. Eng.* **124,** 809–816 (1998).

14. Fung Tat-Ching, Tan Kang-Hai & Lok Tat-Seng. Elastic Constants for Z-Core Sandwich Panels. *J. Struct. Eng.* **120,** 3046–3055 (1994).

15. Baratta, F. I. When is a Beam a Plate? *J. Am. Ceram. Soc.* **64,** C–86 (1981).

16. Ashby, M. F. Overview No. 92: Materials and shape. *Acta Metall. Mater.* **39,** 1025–1039 (1991).

17. Vinson, J. *The Behavior of Sandwich Structures of Isotropic and Composite Materials*. (CRC Press, 1999).
