# Supplementary material for: Nanocardboard as a nanoscale analog of hollow sandwich plates
Source: Nat Commun. 2018 Oct 25;9:4442. doi: 10.1038/s41467-018-06818-6 (PMC6202357; doi:10.1038/s41467-018-06818-6)
Supplement: Supplementary file 3 — Description of Additional Supplementary Files [file 41467_2018_6818_MOESM3_ESM.pdf]

Supplementary Movie 1: Experimental bending and recovery of cantilevers with different geometries in the scanning electron microscope.
