# Supplementary material for: Nanocardboard as a nanoscale analog of hollow sandwich plates
Source: Nat Commun. 2018 Oct 25;9:4442. doi: 10.1038/s41467-018-06818-6 (PMC6202357; doi:10.1038/s41467-018-06818-6)
Supplement: Supplementary file 2 — Peer Review File [file 41467_2018_6818_MOESM2_ESM.pdf]

Editorial Note: This manuscript has been previously reviewed at another journal that is not operating a transparent peer review scheme. This document only contains reviewer comments and rebuttal letters for versions considered at Nature Communications.

REVIEWERS' COMMENTS:

Reviewer #1
No comments to authors.

Reviewer #2 (Remarks to the Author):

I am satisfied with the changes the authors made in response to my previous concerns and believe the paper is well suited for publication in Nature Communications. Great work!
